# Distinct Signaling Pathways Distinguish *in vivo* From *in vitro* Growth in Murine Ovarian Follicle Activation and Maturation

**DOI:** 10.3389/fcell.2021.708076

**Published:** 2021-07-23

**Authors:** Mahboobeh Amoushahi, Karin Lykke-Hartmann

**Affiliations:** ^1^Department of Biomedicine, Aarhus University, Aarhus, Denmark; ^2^Department of Clinical Medicine, Aarhus University, Aarhus, Denmark; ^3^Department of Clinical Genetics, Aarhus University Hospital, Aarhus, Denmark

**Keywords:** transcriptome, primordial follicle, *in vitro*, *in vivo*, oocytes

## Abstract

Women with cancer and low ovarian reserves face serious challenges in infertility treatment. Ovarian tissue cryopreservation is currently used for such patients to preserve fertility. One major challenge is the activation of dormant ovarian follicles, which is hampered by our limited biological understanding of molecular determinants that activate dormant follicles and help maintain healthy follicles during growth. Here, we investigated the transcriptomes of oocytes isolated from dormant (primordial) and activated (primary) follicles under *in vivo* and *in vitro* conditions. We compared the biological relevance of the initial molecular markers of mature metaphase II (MII) oocytes developed *in vivo* or *in vitro*. The expression levels of genes involved in the cell cycle, signal transduction, and Wnt signaling were highly enriched in oocytes from primary follicles and MII oocytes. Interestingly, we detected strong downregulation of the expression of genes involved in mitochondrial and reactive oxygen species (ROS) production in oocytes from primordial follicles, in contrast to oocytes from primary follicles and MII oocytes. Our results showed a dynamic pattern in mitochondrial and ROS production-related genes, emphasizing their important role(s) in primordial follicle activation and oocyte maturation. The transcriptome of MII oocytes showed a major divergence from that of oocytes of primordial and primary follicles.

## Introduction

Laparoscopic ovariectomy and subsequent cryopreservation of ovarian tissue is a standard procedure for female cancer patients undergoing potentially sterilizing chemotherapy and/or radiotherapy. Ovarian tissue cryopreservation is the only available technique for preserving the fertility of women; however, it remains an experimental method. Moreover, laparoscopic ovariectomy has been used to stimulate ovarian follicles for infertility treatment (Kawamura et al., 2013). As a means to optimize the options for these patients, a cell culture system that supports the growth and development of oocytes from the human ovarian cortex has been reported (Telfer, 2019b). However, our knowledge of the *in vitro* activation of dormant follicles is lacking; thus, the *in vitro* growth of follicles is difficult and inefficient. Culturing human follicles is very challenging (Telfer, 2019a). In mice, oocytes grown *in vitro* from primordial follicles have resulted in the production of live offspring (Xu et al., 2006; Wang et al., 2011; Mochida et al., 2013; Higuchi et al., 2015) and, thus, represent a promising translational approach for these patients (Telfer, 2019a). Primordial follicle activation involves major growth of oocytes, associated with the proliferation of the surrounding flattened pregranulosa cells and transformation to cuboidal cells (Rodgers and Irving-Rodgers, 2010). Several studies have identified the PI3K–PTEN–AKT–FOXO3 signaling pathway as the main non-gonadotrophic growth factor signaling pathway that regulates the activation of primordial follicles (Castrillon et al., 2003; Salmena et al., 2008; Ernst et al., 2017, 2018; Maidarti et al., 2019, 2020). Mechanistically, the activation of primordial follicles is triggered by elevated PIP3 levels in the oocyte (Liu et al., 2006), a balance strongly counteracted by PTEN. PTEN inhibits PI3K signaling activation (Jagarlamudi et al., 2009). Similarly, FOXO3a, as a downstream effector, inhibits primordial follicle activation by suppressing this pathway (Castrillon et al., 2003). Despite this molecular knowledge, attempts to use AKT stimulators and PTEN inhibitors were deleterious for follicle quality (Maidarti et al., 2020), and inhibition of PTEN increased DNA damage and reduced the DNA repair response in bovine oocytes (Maidarti et al., 2019). This pharmacological inhibition of PTEN during the *in vitro* development of human follicles revealed that although primordial follicles were activated, their growth was compromised (McLaughlin et al., 2014). The oocyte genome must retain its integrity, which is fundamental for *in vitro* follicle development as a clinical approach for young cancer patients. If primordial follicles are inappropriately activated, DNA and gene expression may be compromised, which could decrease oocyte quality. To obtain high-quality oocytes from *in vitro* development, we must elucidate the molecular mechanisms that underlie this complex activation step of primordial follicles *in vivo* and compare them to those *in vitro*.

Although previous studies have reported that *in vitro* culture systems can closely mimic the *in vivo* environment (Sung and Shuler, 2012; Higuchi et al., 2015; Xiao et al., 2017; Wang et al., 2020) and can generate competent metaphase II (MII) oocytes, the effects on gene expression are unclear. Currently, no study has analyzed and compared the transcriptomic profiles of oocytes derived from *in vitro* and *in vivo* conditions.

In this work, we explored the *in vitro* and *in vivo* growth of ovarian follicles and investigated the morphology and gene expression profiles. We report that the morphology of follicles grown *in vivo* versus *in vitro* was comparable for primordial, primary, and secondary follicles, with equal follicle diameters in the respective stages. Interestingly, despite different growth conditions, we observed no apparent differences in PTEN, FOXO3A, and mTOR levels, nor did we detect differences in reactive oxidative stress levels. Oocytes underwent stage-specific laser-capture microdissection (LCM), and subsequent RNA sequencing revealed distinct transcriptomes for oocytes isolated from primordial and primary follicles. We further established transcriptomic profiles for *in vivo-* and *in vitro*-grown MII oocytes. We observed no differences in the genes encoding PTEN, AKT, PI3K, or mTOR. Interestingly, we observed differential gene expression for other factors, particularly those related to cell cycle regulation, transforming growth factor beta (TGF-β) signaling, signal transduction, Wingless-type mouse mammary tumor virus integration site (Wnt) signaling, and mitochondrial function. These observations showed that while the overall morphology is conserved, molecular differences are detected between oocytes grown *in vivo* compared with *in vitro*, which does not appear to affect oocyte quality. The transcriptomic data can be used to predict molecular targets for primordial follicle activation for infertility treatment for cancer patients and could be useful for middle-aged infertile women and women with premature menopause. These results help elucidate the *in vitro* growth of ovarian follicle cells using the mouse ovary as an experimental model by determining gene expression in oocytes from dormant and activated follicles as well as mature oocytes. We thus describe the initial rate-limiting follicle activation step as well as the end product, the fertile oocyte. During this time, many critically important cellular processes, including activation, differentiation, and the initial events of meiosis, occur. We report expression differences between oocytes from primordial and primary follicles and mature oocytes. These studies illuminate the activation and requirements of ovarian folliculogenesis and provide a resource for analysis of the clinical development and physiology of mammalian ovarian follicle formation.

## Results

### Transcriptome Study Design in Primordial and Primary Follicles

**FIGURE 1:**
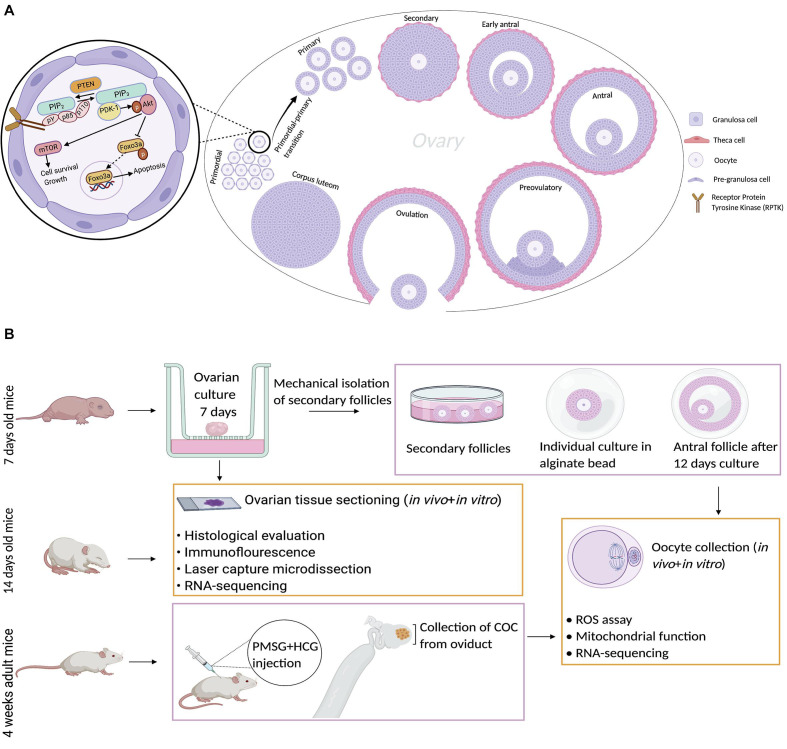
Schematic images representing mammalian ovaries and the experimental design. **(A)** Different developmental stages of follicles are shown from primordial follicles to ovulation and formation of the corpus luteum. The PTEN–AKT–FOXO3 signaling pathway is represented in higher magnification of one primordial follicle, showing its role in the primordial–primary follicle transition. Dimerization and autophosphorylation of RPTK molecules result in the generation of PIP_3_ from PIP_2_. PTEN is a negative regulator of PI_3_K, which converts PIP_3_ to PIP_2_. Akt is activated by binding to PIP_3_ and phosphorylated by PDK1. Activated AKT leads to phosphorylation of Foxo3a and its localization in the cytoplasm, preventing relocation of unphosphorylated Foxo3a to the nucleus to induce apoptosis. Activated AKT can also activate mTOR and induce cell survival and growth. **(B)** The experimental design of the study is shown for both the *in vivo* and *in vitro* groups. Created with BioRender.com.

To assess the morphology of ovarian follicles and ovaries from the *in vivo* and *in vitro* groups, we compared the distinct morphology of primordial, primary, and secondary follicles (Figure 1A). We observed flat pregranulosa cells in the primordial follicles, but they grew and became cuboidal in the primary and secondary stages (Figure 1A). At the end of follicle development, the oocyte progresses to the diplotene stage of prophase I and then undergoes arrest until ovulation. The oocyte resumes meiotic maturation in response to the midcycle luteinizing hormone (LH) surge, generating MII oocytes. We extended our *in vivo* and *in vitro* analysis to include morphological, transcriptomic, and biochemical analyses of metaphase II oocytes. Under our *in vitro* conditions, for final oocyte maturation, human chorionic gonadotropin (hCG) was used to induce ovulation (Figure 1B) and reflect the endogenous LH surge.

### The Development Is Comparable Between the *in vivo* and *in vitro* Groups

**FIGURE 2:**
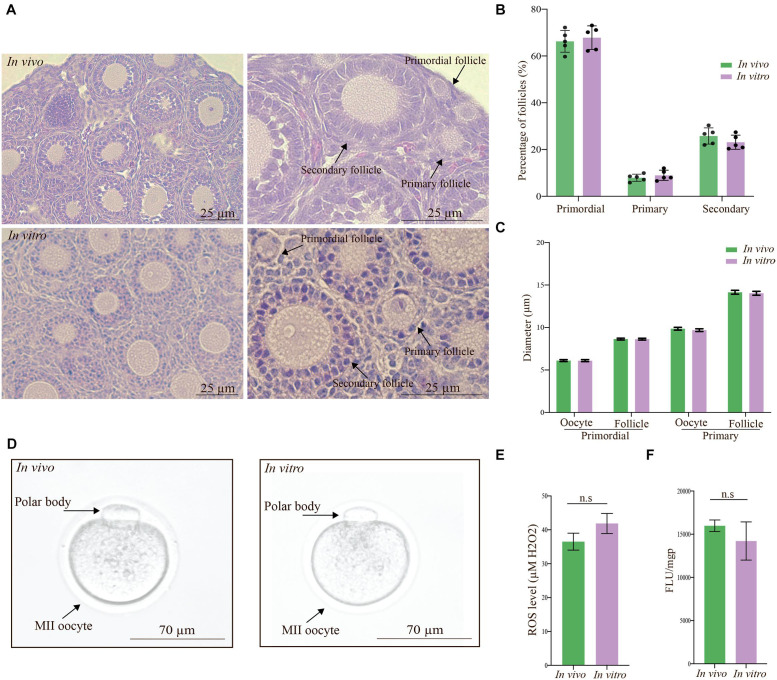
Evaluation of ovaries and metaphase II (MII) oocytes based on morphology, development, and mitochondrial function. **(A)** Photomicrographs show the ovaries in the *in vivo* and *in vitro* groups stained with hematoxylin and eosin at low and high magnifications. The integrity and structural organization of ovaries from the *in vivo* group were similar to those of ovaries from the *in vitro* group. Scale bar: 25 μm. **(B)** The percentage of follicles in each developmental stage was calculated based on the number of normal follicles from the *in vivo* (*n* = 5 ovaries, *n* = 1,314 primordial, *n* = 170 primary, and *n* = 440 secondary follicles) and *in vitro* (*n* = 5 ovaries, *n* = 1,152 primordial, *n* = 134 primary, and *n* = 438 secondary follicles) groups. There was no significant difference between the *in vivo* and *in vitro* groups in each follicle developmental stage. Values are given as the mean ± standard error (SE). **(C)** The diameter of oocytes and follicles in primordial and primary follicle stages. There was no significant difference between the *in vivo* and *in vitro* groups. Values are given as the mean ± SE. **(D)** Representative photomicrographs of MII oocytes from the *in vivo* and *in vitro* groups. Alignment based on the morphology of MII oocytes derived under *in vitro* conditions with *in vivo*-derived MII oocytes was clear. Scale bar: 70 μm. **(E)** Reactive oxygen species (ROS) levels in collected MII oocytes from the *in vivo-* and *in vitro*-matured groups. There was no significant difference between the groups. Values are given as the mean ± SE. **(F)** Uptake of the cationic carbocyanine dye JC-1 into the matrix and mitochondrial membrane potential of collected MII oocytes from the *in vivo-* and *in vitro*-matured groups. There was no significant difference between the groups. Values are given as the mean ± SE.

The growing follicles were well organized with regard to oocyte size and membrane “clearness,” and the morphology of the granulosa cells was consistent in both groups (Figure 2A). The percentage of follicles in each developmental stage was based on the number of follicles observed in equal numbers of ovaries (*n* = 5 in each group) from the *in vivo* group, which included primordial (*n* = 1,314), primary (*n* = 170), and secondary (*n* = 440) follicles, and the *in vitro* group, which included primordial (*n* = 1,152), primary (*n* = 134), and secondary (*n* = 438) follicles. The proportions of normal follicles from the *in vivo* group at the primordial, primary, and secondary stages were 66.28 ± 2.08, 7.93 ± 0.68, and 25.78 ± 1.55, respectively, while the percentages of normal follicles from the *in vitro* group at these stages were 67.85 ± 2.24, 9.00 ± 0.97, and 23.13 ± 1.35%, respectively (Figure 2B). The percentage of normal follicles at different developmental stages was not significantly different between the groups. The integrity and structural organization of ovaries from the *in vivo* group were similar to those from the *in vitro* group after a culture period (data not shown).

To evaluate and compare the growth of the follicles, we measured the follicle and oocyte diameters in the ovaries from the *in vivo* and *in vitro* groups. The mean diameters of oocytes from primordial follicles and primordial follicles in ovaries from the *in vivo* group were 8.64 ± 0.10 μm and 6.11 ± 0.11 μm, respectively, and the corresponding diameters in ovaries from the *in vitro* group were 8.63 ± 0.10 μm and 6.10 ± 0.12 μm, respectively (Figure 2C). In comparison, the mean diameters of oocytes from primary follicles (9.86 ± 0.17 μm) and primary follicles (14.15 ± 0.23 μm) in ovaries from the *in vivo* group were comparable to the corresponding diameters in ovaries from the *in vitro* group (9.69 ± 0.16 μm and 14.03 ± 0.23 μm, respectively) (Figure 2C).

In summary, the morphology of oocytes and follicles from the primordial and primary follicle stages was comparable with regard to growth and size in the *in vivo* and *in vitro* groups.

### The Quality of *in vivo-* and *in vitro*-Grown Mature Oocytes Is Comparable

The morphology of *in vivo*-matured MII oocytes was comparable to that of *in vitro*-matured MII oocytes (Figure 2D), as shown by the shape of the oocytes, which was classical (round with a defined membrane), and the presence of a polar body, indicating healthy *in vitro* growth.

*In vitro* maturation is a popular technology for fertility treatment and preservation. However, *in vitro* culture conditions can increase the reactive oxygen species (ROS) level, which is one of the major causes of compromised embryonic development (Cagnone and Sirard, 2016; Lan et al., 2019; Jamil et al., 2020). Elevated levels of ROS trigger granulosa cell apoptosis, which reduces the transfer of nutrients and survival factors to oocytes, leading to apoptosis. We compared the ROS levels in the *in vivo-*grown MII oocytes with those in the *in vitro*-grown MII oocytes using the 2′,7′-dichlorodihydrofluorescein diacetate (DCFH-DA) fluorescence assay. MII oocytes were collected from both groups and the concentrations of ROS were determined in the MII oocytes from the *in vivo* (36.51 ± 2.51 μM H_2_O_2_) and *in vitro* (41.87 ± 2.97 μM H_2_O_2_) groups (Figure 2E), and no significant difference in ROS levels was observed.

Mitochondrial dysfunction, which has been associated with oocyte quality and an increase in the mitochondrial membrane potential during oocyte maturation, limits current fertility treatments. To determine whether *in vitro* maturation of MII oocytes results in mitochondrial changes, we measured the uptake of the cationic carbocyanine dye JC-1 into the matrix and the mitochondrial membrane potential of collected MII oocytes from the *in vivo-* and *in vitro*-matured groups (Figure 2F). The uptake of JC-1 into the matrix of MII oocytes from the *in vivo* and *in vitro* groups was 15,984 ± 669 and 14,216.33 ± 2,208.58 fluorescence units (FLU)/milligram protein (mgP), respectively, revealing no significant difference between the groups.

### FOXO1, FOXO3a, PTEN, and mTOR Are Similarly Expressed in the *in vivo* and *in vitro* Groups

**FIGURE 3:**
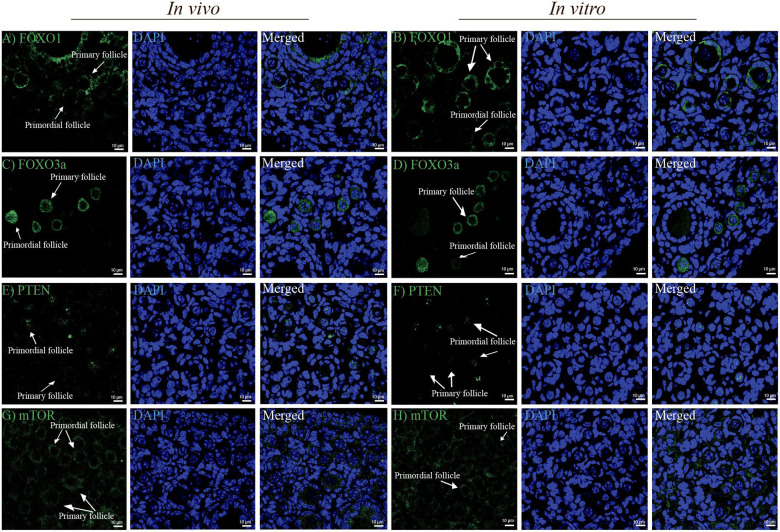
Confocal microscopy images showing the distribution of FOXO1, FOXO3a, PTEN, and mTOR in mouse ovaries from the *in vivo* and *in vitro* groups. **(A,B)** FOXO1 protein was similarly localized in granulosa cells in ovaries from the *in vivo* and *in vitro* groups. **(C,D)** FOXO3a was distributed in the nucleus of primordial follicles and cytoplasm of primary follicles in the *in vivo* and *in vitro* groups. **(E,F)** PTEN was distributed more intensely in oocytes of primordial follicles than in oocytes of primary follicles in the *in vivo* and *in vitro* groups. Notably, PTEN in oocytes of primary follicles was undetectable. **(G,H)** mTOR was equally distributed in oocytes of primordial and primary follicles from both the *in vivo* and *in vitro* groups. DAPI staining shows the cell nuclei. Scale bar: 10 μm.

Next, we examined the level and distribution of known follicle regulators in primordial and primary follicles (Figure 1A) of the *in vivo* group compared with the *in vitro* group. We examined the distribution of FOXO1, FOXO3a, PTEN, and mTOR by immunofluorescence antibody staining of ovarian sections from *in vivo-*grown (Figures 3A,C,E,G) and *in vitro-*grown (Figures 3B,D,F,H) follicles. Immunofluorescence detection of FOXO1 showed that it was specifically distributed in granulosa cells, with no difference between ovaries from the *in vivo* (Figure 3A) and *in vitro* groups (Figure 3B). FOXO3a was distributed according to the follicular stage; in primordial follicles, FOXO3a was enriched in the nucleus, both *in vivo* (Figure 3C) and *in vitro* (Figure 3D). Upon activation, FOXO3a translocates from the nucleus, and thus, in primary follicles, FOXO3a was localized in the cytoplasm, both *in vivo* (Figure 3C) and *in vitro* (Figure 3D). To eliminate the possibility of altered PTEN levels and the associated DNA damage, we investigated the expression of PTEN and found that this important ovarian regulator was expressed at the same level with a similar distribution in oocytes from primordial follicles *in vivo* (Figure 3E) and *in vitro* (Figure 3F). The expression of PTEN in primary follicles was undetectable (Figures 3E,F). We also examined the expression of mTOR and likewise found this protein to be equally expressed and distributed in primordial and primary follicles under *in vivo* (Figure 3G) and *in vitro* (Figure 3H) conditions.

In summary, no significant differences were detected in the tested protein levels or distribution between *in vivo* and *in vitro* in primordial and primary follicles (Supplementary Figure 1).

### *In vivo* and *in vitro* Oocyte Transcriptome Profiling Suggest Extensive Gene Expression in Cell Proliferation and Growth-Related Gene Pathways

**FIGURE 4:**
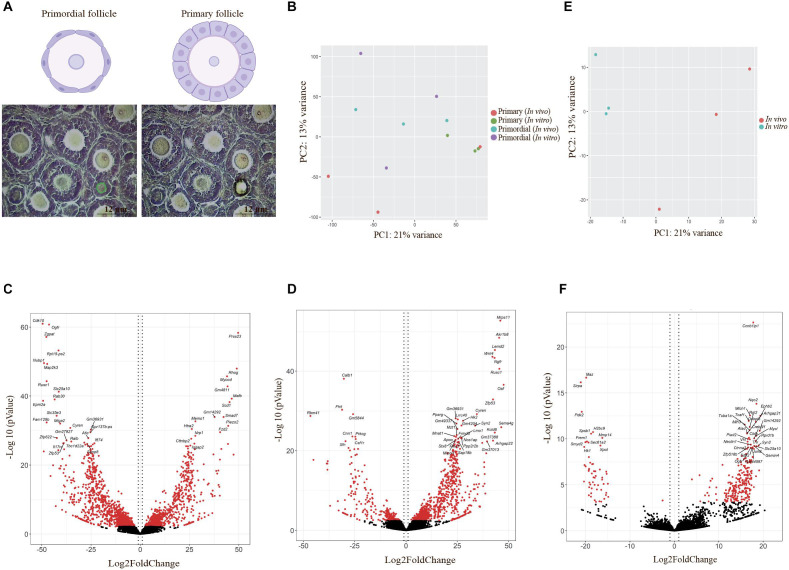
Laser capture microdissection and differentially expressed gene (DEG) visualization. **(A)** Schematic images represent follicles in primordial and primary follicles. Primordial follicles are shown with flattened pregranulosa cells surrounding the oocyte, and primary follicles are shown with a single layer of cuboidal granulosa cells. Photomicrographs showing precise determination and cutting by laser capture microdissection based on defined classification of different developmental stages of follicles in the study. Scale bar: 12 μm. **(B)** Principal component analysis (PCA) of DEGs in oocytes from primordial and primary follicles in the *in vivo* and *in vitro* groups, generated by R. Red and green dots show oocytes from primary follicles in the *in vivo* and *in vitro* groups, respectively. Blue and purple dots define oocytes from primordial follicles in the *in vivo* and *in vitro* groups, respectively. **(C,D)** Volcano plot of DEGs in oocytes from primordial **(C)** and primary follicles **(D)** in the *in vitro* group compared with the *in vivo* group. Vertical dotted lines indicate ± 1 log2 fold change. The 20 most significant genes are shown in volcano plots. Red dots indicate significantly expressed genes with adjusted *p*-values < 0.05, and black dots indicate non-significantly expressed genes. **(E)** PCA of DEGs in the *in vivo- and in vitro*-grown mature oocytes plotted using R. Red and blue dots represent *in vivo-* and *in vitro*-grown mature oocytes, respectively. **(F)** Volcano plot of DEGs in MII oocytes in the *in vitro* and *in vivo* groups. Vertical dotted lines indicate ± 1 log2 fold change. The 20 most significant genes are shown in volcano plots. Red dots indicate significantly expressed genes with adjusted *p*-values < 0.05, and black dots indicate non-significantly expressed genes.

Oocytes were isolated by stage-specific LCM from follicles and categorized into three replicates for each stage from the *in vivo* (*n* = 277 primordial stage, *n* = 170 primary stage) and *in vitro* (*n* = 299 primordial stage, *n* = 250 primary stage) groups (12 samples in total) (Figure 4A). RNA sequencing was performed per sample, and mapping to the mouse genome (mm10) was performed with RefSeq genes as the template. The normalization of expression values to achieve transcripts per million (TPM), lfcSE, *p*-values, and adjusted *p*-values was performed using the DESeq2 R package (Love et al., 2014). Visualization of principal component analysis (PCA) was performed, and significantly differentially expressed genes (SDEGs) (gene abbreviations are listed in Table 1) and volcano plots were generated.

**TABLE 1:** List of gene names and gene abbreviations.

Gene abbreviations	Definitions
*Aamdc*	Adipogenesis associated mth938 domain containing
*Abcc4*	ATP binding cassette subfamily C member 4
*Acot2*	Acyl-CoA thioesterase 2
*Akr1b8*	Aldo-Keto reductase family 1 member B8
Akt/Pkb	Protein kinase B
*Ankrd23*	Ankyrin repeat domain 23
*Ap3m2*	Adaptor related protein complex 3 subunit Mu 2
*Arl5b*	ADP ribosylation factor like GTPase 5B
*Arsi*	Arylsulfatase family member I
*Atp4a*	ATPase H+/K+ transporting subunit alpha
*Barx2*	Homeobox protein BarH-like 2
*Bend7*	BEN domain containing 7
*Btbd11*	BTB domain containing 11
*Btbd8*	BTB domain containing 8
*Cacna1c*	Calcium voltage-gated channel subunit alpha1 C
*Car 14*	Carbonic anhydrase 14
*Ccnblip1*	Cyclin B1 interacting protein 1
Cdk10	Cyclin dependent kinase 10
*Cenpo*	Centromere protein O
*Chrm4*	Cholinergic receptor muscarinic 4
*Clec2l*	C-type lectin domain family 2 member L
*Cmtm*	CKLF like MARVEL transmembrane
*Cnn1*	Calponin 1
*Coq3*	Coenzyme Q3, methyltransferase
*Cpne8*	Copine 8
*Cpxm2*	Carboxypeptidase X, M14 family member 2
*Crip3*	Cysteine rich protein 3
*Cybrd1*	Cytochrome B reductase 1
Cyren	Cell cycle regulator of NHEJ
*Dcxr*	Dicarbonyl and L-xylulose reductase
*Ddrl*	Discoidin domain receptor tyrosine kinase 1
*Depdc1a*	DEP domain containing 1
*Dnajc24*	DnaJ heat shock protein family (Hsp40) member C24
*Dnd1*	DND microRNA-mediated repression inhibitor 1
*Dpep3*	Dipeptidase 3
*Ephb2*	Ephrin type-B receptor 2
*Epm2a*	Epilepsy, progressive myoclonus type 2A
*Fbxo32*	F-box protein 32
*Fgf7*	Fibroblast growth factor 7
*Fgf9*	Fibroblast growth factor 9
Foxo3	Forkhead box O3
*Foxred1*	FAD dependent oxidoreductase domain containing 1
*Fzd2*	Frizzled class receptor 2
*Fzd2*	Frizzled class receptor 2
*Gadd45a*	Growth arrest and DNA damage inducible alpha
*Gadd45g*	Growth arrest and DNA damage inducible gamma
*Galnt7*	Polypeptide *N*-acetylgalactosaminyltransferase 7
*Gas1*	Growth arrest specific 1
*Gemin2*	Gem nuclear organelle associated protein 2
*Gstm4*	Glutathione S-transferase Mu 4
*Gstm6*	Glutathione S-transferase Mu 6
*Gucy2c*	Guanylate cyclase 2C
*Hint3*	Histidine triad nucleotide binding protein 3
*Hk2*	Hexokinase 2
*Hmgcl*	3-hydroxymethyl-3-methylglutaryl-coenzyme A lyase
*Il17ra*	Interleukin 17 receptor A
*Ildr2*	Immunoglobulin like domain containing receptor 2
*Jund*	Jund proto-oncogene, AP-1 transcription factor subunit
*KIM15*	Kelch like family member 15
*Krt36*	Keratin 36
*Krt7*	Keratin 7
*Lctl*	Lactase like
*Lemd2*	LEM domain nuclear envelope protein 2
*Leprotl1*	Leptin receptor overlapping transcript like 1
*Lrrc40*	Leucine rich repeat containing 40
*Lypd6b*	LY6/PLAUR domain containing 6B
*Mageh1*	Melanoma-associated antigen H1
*Map2k3*	Mitogen-activated protein kinase kinase 3
*Mef2c*	Myocyte enhancer factor 2C
*Mgat3*	Beta-1,4-mannosyl-glycoprotein 4-beta-*N*-acetylglucosaminyltransferase
*Miga2*	Mitoguardin 2
*Mlh3*	Mutl homolog 3
*Mnat1*	Menage A trois 1
*Mocs1*	Molybdenum cofactor synthesis 1
*Mrps11*	Mitochondrial ribosomal protein S11
*Mt2*	Melatonin receptor 1B
Mtch1	Mitochondrial carrier homolog 1
Mtor	Mechanistic target of rapamycin kinase
*Mycl*	MYCL proto-oncogene, BHLH transcription factor
*Mycn*	MYCN proto-oncogene, BHLH transcription factor
*Myocd*	Myocardin
*Nabp2*	Nucleic acid binding protein 2
*Naif1*	Nuclear apoptosis inducing factor 1
*Ndufs7*	NADH: ubiquinone oxidoreductase core subunit S7
Ngfr	Nerve growth factor receptor
*Niban2*	Niban apoptosis regulator 2
*Nox4*	NADPH oxidase 4
*Nqo2*	*N*-ribosyldihydronicotinamide: Quinone reductase 2
*Nr3c1*	Nuclear receptor subfamily 3 group C member 1
*Nrn1*	Neuritin 1
*Nubp1*	Nucleotide binding protein 1
*Oaf*	Out at first homolog
Ogfr	Opioid growth factor receptor
*Oma1*	Overlapping with the M-AAA protease 1 homolog
*Pamr1*	Peptidase domain containing associated with muscle regeneration 1
*Pcdhb20*	Protocadherin beta 20
*Pdk2*	Pyravate dehydrogenase kinase 2
*Pdlim2*	PDZ and LIM domain 2
*Phf7*	PHD finger protein 7
Pi3k	Phosphatidylinositol 3-kinases
Pip3	Phosphate-inositol (3,4,5)-triphosphate
*Pitx2*	Paired-like homeodomain transcription factor 2
*Piwil2*	Piwi like RNA-mediated gene silencing 2
*Pkia*	CAMP-dependent protein kinase inhibitor alpha
*Pmsg1*	Proteasome assembly chaperone 1
*Ppplr3e*	Protein phosphatase 1 regulatory subunit 3E
*Prdx4*	Peroxiredoxin 4
*Prkg1*	Protein kinase CGMP-dependent 1
Psmg2	Proteasome assembly chaperone 2
Pten	Phosphatase and tensin homolog
*Ptpro*	Protein tyrosine phosphatase receptor type O
*Pura*	Purine rich element binding protein A
Ralb	RAS like proto-oncogene B
*Rhog*	Ras homolog family member G
*Rnf217*	Ring finger protein 21
*Rps6ka2*	Ribosomal protein S6 kinase A2
*Rsc1a1*	Regulatory solute carrier protein, family 1, member 1
*Scd1*	Stearoyl-CoA desaturase 1
*Sgk1*	Serum/glucocorticoid regulated kinase 1
*Sh3d21*	SH3 domain containing 21
*Shisa4*	Shisa family member 4
*Slc25a10*	Solute carrier family 25 member 10
*Slc25a13*	Solute carrier family 25 member 13
*Slc35e3*	Solute carrier family 35 member E3
*Smad6*	Mothers against decapentaplegic homolog 6
*Smad7*	Mothers against decapentaplegic homolog 7
*Smim12*	Small integral membrane protein 12
*Snai2*	Snail family transcriptional repressor 2
*Snapc3*	Small nuclear RNA activating complex polypeptide 3
*Snx25*	Sorting nexin 25
*Sox11*	SRY-box transcription factor 11
*Stc1*	Stanniocalcin 1
*Syn2*	Synapsin II
*Tcaim*	T cell activation inhibitor, mitochondrial
*Th*	Tyrosine hydroxylase
*Timm10*	Translocase of inner mitochondrial membrane 10
*Timm21*	Translocase of inner mitochondrial membrane 21
*Timm8a1*	Translocase of inner mitochondrial membrane 8A
*Tmc7*	Transmembrane channel like 7
*Tomm40l*	Translocase of outer mitochondrial membrane 40 like
*Tph2*	Tryptophan hydroxylase 2
*Tpm2*	Tropomyosin 2
*Traf2*	TNF receptor associated factor 2
*Uqcrfs1*	Ubiquinol-cytochrome C reductase, rieske iron-sulfur polypeptide 1
*Vamp3*	Vesicle associated membrane protein 3
*Wnt2b*	Wingless-type MMTV integration site family, member 2B
*Wnt4*	Wingless-type MMTV integration site family, member 4
*Zc3h8*	Zinc finger CCCH-type containing 8
*Zfp438*	Zinc finger protein 438

Principal component analysis indicated variation in expression values and different expression patterns in oocytes of primordial and primary follicles from the *in vivo* and *in vitro* groups (Figure 4B). However, clustering of the genes between oocytes of primordial and primary follicles from the *in vivo* and *in vitro* groups was not clear (Figure 4B), revealing variability between samples in each developmental stage and group. For the identification of SDEGs with large fold changes, volcano plots were generated. We selected the top 20 significant genes in oocytes of primordial (Figure 4C) and primary follicles (Figure 4D) from the *in vitro* group compared with the *in vivo* group. Interestingly, in oocytes from primordial follicles (Figure 4C), the levels of cell proliferation- and growth-related genes such as *Cdk10*, *Ogfr*, *Cyren*, and *Ralb* and *Psmg2*, *Pkia*, *Gadd45g*, *Nubp1*, and *Niban2* were highly downregulated. Moreover, mitogen-activated protein kinase (MAPK) pathway-involved genes, such as *Map2k3*, and mitochondrial genes, such as *Slc25a10* and *Miga2*, were among the top 20 significantly downregulated genes. Furthermore, the levels of the ROS-related genes *Dcxr* and *Foxred1* were notably downregulated in the *in vivo* to *in vitro* comparison. Additionally, the levels of the Wingless-type mouse mammary tumor virus integration site (Wnt) signaling pathway genes *Wnt2b* and *Mgat3* were highly downregulated. Among the top 20 upregulated genes in oocytes from primordial follicles, *Smad7*, which is involved in transforming growth factor beta (TGF-β) signaling, was identified. *Fzd2*, which is also involved in Wnt signaling, was among the top 20 upregulated genes in oocytes from primordial follicles (Table 2).

**TABLE 2:** Top most significant DEGs in oocytes from primordial follicles between *in vivo* and *in vitro* groups.

Gene name	Description	Log2FC	*p*-Value	TPM	Up/Down Regulated	Function
*Cdk10*	Cyclin-dependent kinase 10	–49.56	1.04E-61	9.67	Down	Cell cycle
*Ogfr*	Opioid growth factor receptor-like protein	-46.32	1.93E-61	12.11	Down	
*Cyren*	Cell cycle regulator of non-homologous end joining	–35.54	1.77E-31	0.47	Down	
*Ralb*	RAS Like Proto-Oncogene B	–34.99	1.48E-27	0.82	Down	
*Psmg2*	Proteasome Assembly Chaperone 2	–38.92	8.71E-13	5.91	Down	
*Pura*	Purine Rich Element Binding Protein A	–26.19	9.33E-23	18.77	Down	TGF-beta signaling
*Snx25*	Sorting Nexin 25	–19.64	3.95E-15	4.48	Down	
*Smad7*	SMAD Family Member 7	42.25	1.12E-34	10.38	Up	
*Wnt2b*	Wnt Family Member 2	–40.34	1.85E-20	13.30	Down	Wnt signaling
*Wnt5a*	Wnt Family Member 5A	–20.62	5.60E-05	5.18E-07	Down	
*Mgat3*	Beta-1,4-Mannosyl-Glycoprotein 4-Beta-*N*-Acetylglucosaminyltransferase	–33.28	5.63E-14	18.44	Down	
*Fzd1*	Frizzled Class Receptor 1	–10.76	0.002	25.61	Down	
*Fzd2*	Frizzled Class Receptor 2	40.55	2.94E-30	7.35	Up	
*Map2k3*	Mitogen-Activated Protein Kinase Kinase 3	–47.27	4.65E-50	13.18	Down	Signal transduction
*Slc25a10*	Solute Carrier Family 25 Member 10	–41.37	5.35E-42	5.13	Down	Mitochondria
*Miga2*	Mitoguardin 2	–40.64	9.23E-33	5.30	Down	
*Timm21*	Translocase of Inner Mitochondrial Membrane 21	–24.19	3.32E-21	76.91	Down	
*Timm8a1*	Translocase of Inner Mitochondrial Membrane 8A	–19.01	3.57E-09	11.46	Down	
*Dcxr*	Dicarbonyl and L-Xylulose Reductase	–39.55	1.66E-22	2.70	Down	ROS signaling
*Foxred1*	FAD Dependent Oxidoreductase Domain Containing 1	–34.28	1.10E-09	0.83	Down	
*Nox4*	NADPH Oxidase 4	–33.39	3.60E-12	0.58	Down	
*Zgpat*	Zinc finger CCCH-type with G patch domain-containing protein	–47.742685	7.38E-58	1.45517695	Down	Transcription
*Gstm4*	Glutathione *S*-transferase Mu 4	–33.60	1.02E-10	40.25	Down	Metabolism
*Abcd2*	ATP-binding cassette sub-family D member 2	30.056805	1.18E-06	1.50857192	Up	Transport

In oocytes from primary follicles (Figure 4D), the levels of *Mnat1*, *Cyren*, and *Psmg2*, which are associated with cell cycle processes, were highly upregulated. Notably, genes involved in the TGF-β signaling pathway, such as *Smad6*, *Pitx2*, and *Ngfr*, were also in the top 20 significantly upregulated genes. In addition, the mitochondria-related gene *Mrps11* and the ROS-related gene *Akr1b8* were in the top 20 significantly upregulated genes in oocytes from primary follicles. Interestingly, *Wnt4*, which is involved in the Wnt signaling pathway, and *Lemd2*, which is involved in the MAPK signal transduction pathway, were also among the top 20 significantly upregulated genes in oocytes from primary follicles (Table 3).

**TABLE 3:** Top most significant DEGs in oocytes from primary follicles between *in vivo* and *in vitro* groups.

Gene name	Description	Log2FC	*p*-Value	TPM	Up/Down Regulated	Function
*Cyren*	Cell Cycle Regulator Of NHEJ	34.54	6.25E-30	0.47	Up	Cell cycle
*Psmg2*	Proteasome Assembly Chaperone 2	38.31	1.95E-12	5.91	Up	
*Mnat1*	MNAT1 Component Of CDK Activating Kinase	23.52	2.16E-24	11.42	Up	
*Ngfr*	Nerve Growth Factor Receptor	43.16	4.66E-44	3.25	Up	TGF-beta signaling
*Smad6*	SMAD Family Member 6	23.83	8.59E-10	22.09	Up	
*Fgf9*	Fibroblast Growth Factor 9	15.86	8.04E-09	11.91	Up	
*Pitx2*	Paired Like Homeodomain 2	25.66	3.15E-12	3.41	Up	
*Wnt4*	Wnt Family Member 4	42.14	2.56E-44	9.92	Up	Wnt signaling
*Lgr6*	Leucine Rich Repeat Containing G Protein-Coupled Receptor 6	11.24	4.85E-06	6.88	Up	
*Rnf43*	Ring Finger Protein 43	11.11	0.00	3.07	Up	
*Lemd2*	LEM domain-containing protein 2	43.32	4.81E-46	3.01	Up	Signal transduction
*Plpp7*	Inactive phospholipid phosphatase 7	30.91	5.07E-09	1.97	Up	Signaling
*Hhip*	Hedgehog-interacting protein	31.50	1.48E-10	3.40	Up	Signaling
*Timm21*	Translocase Of Inner Mitochondrial Membrane 21	19.94	8.11E-15	76.91	Up	Mitochondria
*Mrps11*	Mitochondrial Ribosomal Protein S11	45.96	2.04E-53	2.37	Up	
*Tomm40l*	Mitochondrial import receptor subunit TOM40B	30.84	8.69E-10	7.44	Up	
*Endog*	Endonuclease G, mitochondrial	–22.81	5.83E-08	4.87	Down	
*Akr1b8*	Aldo-Keto Reductase Family 1 Member B10	45.38	4.11E-49	9.28	Up	ROS signaling
*Ptger3*	Prostaglandin E2 receptor EP3 subtype	–23.92	8.59E-20	4.19	Down	Receptor
*Soxl8*	Transcription factor SOX-18	38.33	7.41E-15	31.79	Up	Transcription
*Ogfrl1*	Opioid growth factor receptor-like protein 1	17.34	6.43E-13	2.52	Up	Growth factor
Cybrd1	Cytochrome B Reductase 1	38.16	1.29E-16	0.48	Up	
*Slc35d2*	UDP-*N*-acetylglucosamine/ UDP-glucose/ GDP-mannose transporter	–28.00	1.95E-21	7.27	Down	
*Gstm4*	Glutathione *S*-transferase Mu 4	23.79	5.02E-06	40.25	Up	Transferase
*Abcd2*	ATP-binding cassette sub-family D member 2	34.04	3.63E-08	1.50	Up	Transport

### The Level of ROS-Related and Mitochondrial Targeted Genes Is Upregulated in MII Oocytes During *in vitro* Growth

To assess the endogenous quality of *in vitro*-grown mature oocytes compared with *in vivo-*matured oocytes, we further identified molecular characteristics by bioinformatic analysis. Thus, *in vivo*-grown oocytes were collected from hormone-stimulated adult mice, and *in vitro*-grown oocytes were collected from two-step-cultured ovaries of neonatal mice and pooled into three replicates for the *in vivo* (*n* = 10 per tube) and *in vitro* (*n* = 10 per tube) groups (six samples in total) (Figure 1B). RNA sequencing was performed per sample, and mapping to the mouse genome (mm10) was performed with RefSeq genes as the template. The normalization of expression values to achieve TPM, lfcSE, *p*-values, and adjusted *p*-values was performed using the DESeq2 R package. For identification of variation in expression values in MII oocytes from the *in vivo* and *in vitro* groups, a PCA plot was generated, which showed different clustering between the *in vivo* and *in vitro* groups (Figure 4E), suggesting a different gene expression pattern between *in vivo-* and *in vitro*-grown MII oocytes. Visualization of genes with large fold changes in volcano plots showed the top 20 significantly differentially expressed genes in MII oocytes from the *in vivo* and *in vitro* groups (Figure 4F). The cell cycle-related genes *Ccnb1ip1* and *Piwil2* were among the top 20 downregulated genes. Moreover, the MAPK signaling-related genes *Jund* and *Ephb2* were among the top 20 upregulated genes. Surprisingly, *Nqo2*, which is involved in the regulation of ROS, and mitochondrial targeted genes such as *Mtch1* and *Slc25a10* were also among the top 20 upregulated genes in MII oocytes (Table 4).

**TABLE 4:** Top most significant DEGs in MII oocytes between *in vivo* and *in vitro* groups.

Gene name	Description	Log2FC	*p*-Value	TPM	Up/Down Regulated	Function
*Ccnblip1*	Cyclin B1 Interacting Protein 1	17.76	2.14E-23	15.89	Up	Cell cycle
*Mlh3*	MutL Homolog 3	16.92	1.14E-11	2.99	Up	
*Ralb*	RAS Like Proto-Oncogene B	14.94	1.80E-06	0.82	Up	
*Cyren*	Cell Cycle Regulator Of NHEJ	16.95	2.06E-08	0.47	Up	
*Piwil2*	Piwi Like RNA-Mediated Gene Silencing 2	16.55	1.04E-10	11.14	Up	
*Ephb2*	Developmentally Regulated Eph-Related Tyrosine Kinase	19.36	3.19E-13	5.76	Up	Signal transduction
*Jund*	JunD Proto-Oncogene, AP-1 Transcription Factor Subunit	17.61	8.52E-10	192.39	Up	
*Map2k3*	Mitogen-Activated Protein Kinase Kinase 3	13.04	4.60E-05	13.18	Up	
*Dixdc1*	DIX Domain Containing 1	17.64	9.89E-09	3.09	Up	Wnt signaling
*Fzd2*	Frizzled Class Receptor 2	15.38	7.32E-06	7.35	Up	
*Snai2*	Snail Family Transcriptional Repressor 2	13.45	0.00	78.26	Up	
*Tpcn1*	Two Pore Segment Channel 1	15.21	7.20E-09	32.63	Up	Calcium signaling
*Slc8a2*	Solute Carrier Family 8 Member A2	15.52	0.00	1.49	Up	
*Kcna1*	Potassium Voltage-Gated Channel Subfamily A Member 1	19.08	2.69E-07	4.58	Up	
*Mtch1*	Mitochondrial Carrier 1	17.04	2.83E-13	103.95	Up	Mitochondria
*Ndufs7*	NADH: Ubiquinone oxidoreductase core subunit S1	16.15	4.52E-09	9.46	Up	
*Mrps11*	Mitochondrial Ribosomal Protein S11	15.87	1.31E-07	2.37	Up	
*Slc25a10*	Solute Carrier Family 25 Member 10	18.45	6.52E-10	5.13	Up	
*Scd1*	Stearoyl-CoA Desaturase	16.55	1.90E-06	3.76	Up	
*Wdr35*	WD Repeat Domain 35	16.94	9.39E-05	2.51	Up	

### Comparison Reveals Distinct Stage-Specific Molecular Characteristics

**FIGURE 5:**
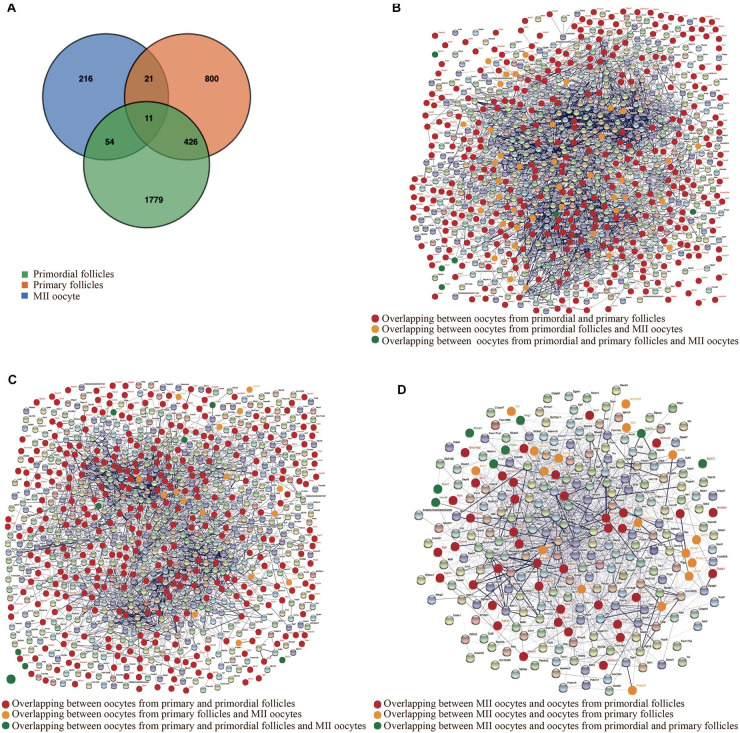
Identification of overlapping significantly differentially expressed genes (SDEGs). **(A)** Venn diagram representing the overlapping SDEGs between oocytes from primordial and primary follicles and metaphase II (MII) oocytes in the *in vitro* and *in vivo* groups. Green, orange, and blue colors show SDEGs of oocytes from primordial follicles, primary follicles, and MII oocytes, respectively. **(B)** Constructed protein–protein interaction network (PPI) from SDEGs of oocytes from primordial follicles in the *in vitro* and *in vivo* groups. Red nodes represent the encoded proteins from overlapping SDEGs between oocytes from primordial and primary follicles in the *in vitro* and *in vivo* groups. Yellow nodes show encoded proteins from overlapping SDEGs between oocytes from primordial follicles and MII oocytes. Green nodes refer to encoded proteins from overlapping SDEGs between oocytes from all developmental stages. **(C)** PPI from SDEGs of oocytes from primary follicles in the *in vitro* and *in vivo* groups. Red nodes represent the encoded proteins from overlapping SDEGs between oocytes from primordial and primary follicles in the *in vitro* and *in vivo* groups. Yellow nodes show encoded proteins from overlapping SDEGs between oocytes from primary follicles and MII oocytes. Green nodes refer to encoded proteins from overlapping SDEGs between oocytes from all developmental stages. **(D)** PPI from SDEGs of MII oocytes in the *in vitro* compared with the *in vivo* groups. Red nodes represent the encoded proteins from overlapping SDEGs between MII oocytes and oocytes from primordial follicles in the *in vitro* and *in vivo* groups. Yellow nodes show encoded proteins from overlapping SDEGs between MII oocytes and oocytes from primary follicles in the *in vitro* and *in vivo* groups. Green nodes refer to encoded proteins from overlapping SDEGs between oocytes from all developmental stages.

Interestingly, despite the different gene expression patterns, overlapping genes between oocytes from primordial and primary follicles were identified. The determination of the interaction between the proteins encoded by SDEGs that overlap between oocytes from primordial and primary follicles could help elucidate the molecular mechanism underlying the *in vivo* and *in vitro* growth of oocytes from primordial and primary follicles. Interestingly, 437 overlapping SDEGs were identified when comparing oocytes from primordial and primary follicles in the *in vivo* and *in vitro* groups. However, when we included SDEGs from MII oocytes, the comparison revealed only 11 overlapping SDEGs for oocytes in the three developmental stages (Figure 5A).

For a better understanding of the potential molecular mechanism during different developmental stages of oocytes, in relation to growth conditions, we mapped the interactions by protein–protein interaction networks (PPIs) of the anticipated protein products encoded by overlapping SDEGs (Figure 5A), between MII oocytes and oocytes from primordial and primary follicles in the *in vivo* and *in vitro* groups (Figures 5B–D and see also Supplementary Figures 2–4).

*Tph2*, *Sgk1*, *Traf2*, *Mycn*, *Hk2*, *Snapc3*, *Gucy2c*, *Mnat1*, *Cenpo*, *Gadd45a*, *Nr3c1*, *Hlcs*, *Nabp2*, *Zc3h8*, *Timm10*, *Tomm40l*, *Slc25a13*, *Uqcrfs1*, *Coq3*, *Acot2*, *Th*, *Prkg1*, *Pmsg1*, *Vamp3*, and *Fgf7* were shown to be highly interconnected in both oocytes from primordial and primary follicles, suggesting a similar pattern for high-grade functional interplay. Moreover, *Pamr1*, *Pcdhb20*, *Hint3*, *Krt36*, *Car14*, *Gstm4*, *Naif1*, *Tmc7*, and *Pdlim2* were slightly interconnected, and *Snx25*, *Ptpro*, *Shisa4*, *Smim12*, *Zfp438*, *Cpxm2*, *Tcaim*, *Sh3d21*, *Crip3*, *Bend7*, *Btbd8*, *Clec2l*, *Phf7*, *Lrrc40*, *Abcc4*, and *Barx2* were visualized with no connections in oocytes from both primordial and primary follicles, indicating the same order of low-grade functional interrelations. In contrast, *Mocs1*, *Lypd6b*, *Hmgcl*, *Klhl15*, *Rsc1a1*, *Ap3m2*, *Dpep3*, *Dnd1*, *Lctl*, *Cnn1*, *Arl5b*, *Ankrd23*, and *Psmg2* had higher interconnections in oocytes from primordial follicles than oocytes from primary follicles, while *Timm21*, *Timm8a1*, *Arsi*, *Mt2*, *Mageh1*, *Gas1*, *Cpne8*, *Leprotl1*, *Cmtm4*, *Ddr1*, *Il17ra*, *Oma1*, and *Aamdc* had higher interrelations in oocytes from primary follicles than oocytes from primordial follicles (Figures 5B,C).

*Btbd11*, *Epm2a*, *Ppp1r3e*, *Rps6ka2*, *Dcxr*, *Pdk2*, *Chrm4*, *Fzd2*, and *Cacna1c* were highly interconnected in oocytes from primordial follicles, while these SDEGs had slight interconnections in MII oocytes. Moreover, *Ogfr*, *Krt7*, *Cdk10*, and *Slc35e3* were determined to be slightly interconnected in both oocytes from primordial follicles and MII oocytes. In contrast, *Nrn1*, *Stc1*, and *Mycl* were highly interconnected in oocytes from primordial follicles, while these SDEGs had slight connections in MII oocytes. Interestingly, *Ralb*, *Fbxo32*, *Map2k3*, *Tpm2*, *Myocd*, *Rhog*, *Snai2*, *Mef2c*, *Nox4*, *Scd1*, and *Rnf217* were determined to be highly interrelated in both MII oocytes and oocytes from primordial follicles (Figures 5B,D).

*Depdc1a* was highly interrelated in oocytes from primary follicles, while it showed a slight connection in MII oocytes. Surprisingly, *Ngfr*, *Syn2*, and *Smad6* were highly interrelated in both MII oocytes and oocytes from primary follicles. Moreover, *Sox11*, *Atp4a*, *Galnt7*, *Lemd2*, *Dnajc24*, *Gemin2*, *Gstm6*, *Ildr2*, and *Oaf* were determined to be slightly interrelated, and *Gm27027* showed no connections in either MII oocytes or oocytes from primary follicles (Figures 5C,D). These results suggested that although SDEGs in MII oocytes showed higher overlap with those in oocytes from primordial follicles, the interconnection of the proteins encoded by overlapping SDEGs between MII oocytes and oocytes from primary follicles was more similar than that of overlapping SDEGs between MII oocytes and oocytes from primordial follicles in the *in vivo* and *in vitro* groups.

In summary, screening of SDEGs by PPI networks showed that most of them had mutual interrelations. Moreover, identification of overlapped SDEGs between oocytes of different developmental stages in PPI showed that despite this overlapping, the interconnections between different developmental stages are different. Therefore, screening of different interconnections in overlapped SDEGs between different developmental follicle stages is fundamental to understand the underlying molecular mechanism from early to late stages of follicular development. Future work is needed to confirm the specific roles of the gene products.

**FIGURE 6:**
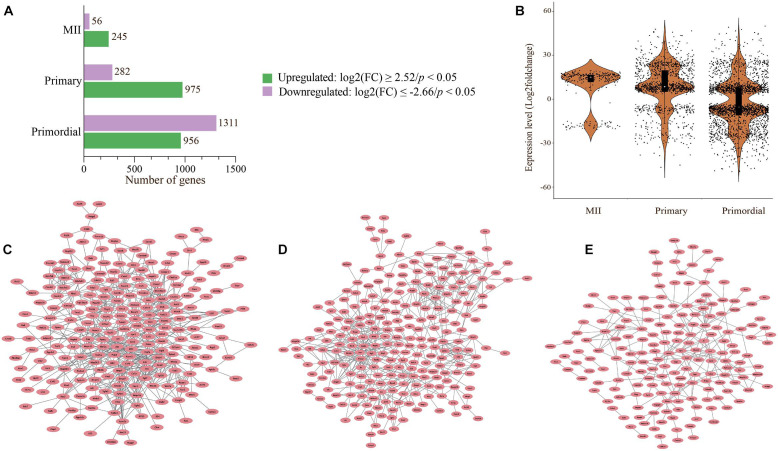
Visualization of SDEGs by bar chart, violin plot, and cloud networks. **(A)** The number of SDEGs (adjusted *p*-value < 0.05) represented by the bar chart. Differential expression analysis and normalization were performed using DESeq2 in R. **(B)** Violin plot representing the distribution of SDEGs in oocytes from primordial and primary follicles and MII oocytes in the *in vivo* and *in vitro* groups. Each dot shows the log2 fold change values in oocytes. **(C–E)** Network of 302 selected SDEGs in oocytes from primordial **(C)** and primary **(D)** follicles and MII oocytes **(E)** in the *in vivo* and *in vitro* groups. Different expression patterns are shown for the *in vivo-* and *in vitro*-grown MII oocytes and oocytes from primordial and primary follicles.

To further investigate the SDEGs, we visualized all SDEGs based on both the number of genes and an absolute fold change of 2 in oocytes from different developmental stages between the *in vivo* and *in vitro* groups (Figures 6A,B). The list of all SDEGs had 2,270 significant genes with 959 upregulated genes and 1,311 downregulated genes in oocytes of primordial follicles. Moreover, the expression levels of 975 and 282 genes were upregulated and downregulated, respectively, in oocytes of primary follicles (Figure 6A). Interestingly, the expression of most of the genes was downregulated in oocytes from primordial follicles but upregulated in oocytes from primary follicles (Figure 6B). Surprisingly, the list of all SDEGs showed that the levels of most of the genes in MII oocytes were upregulated (245 genes), which was also similar to oocytes from primary follicles (Figures 6A,B).

For visualization, 302 selected SDEGs were applied to construct PPI networks (Figures 6C–E). The PPI networks of oocytes from primordial and primary follicles showed different expression patterns of SDEGs in these oocytes between the *in vivo* and *in vitro* groups (Figures 6C,D). Surprisingly, 54 SDEGs showed overlap between MII oocytes and oocytes from primordial follicles, and 21 overlapping SDEGs were detected between MII oocytes and oocytes from primary follicles (Figures 5A,D).

**FIGURE 7:**
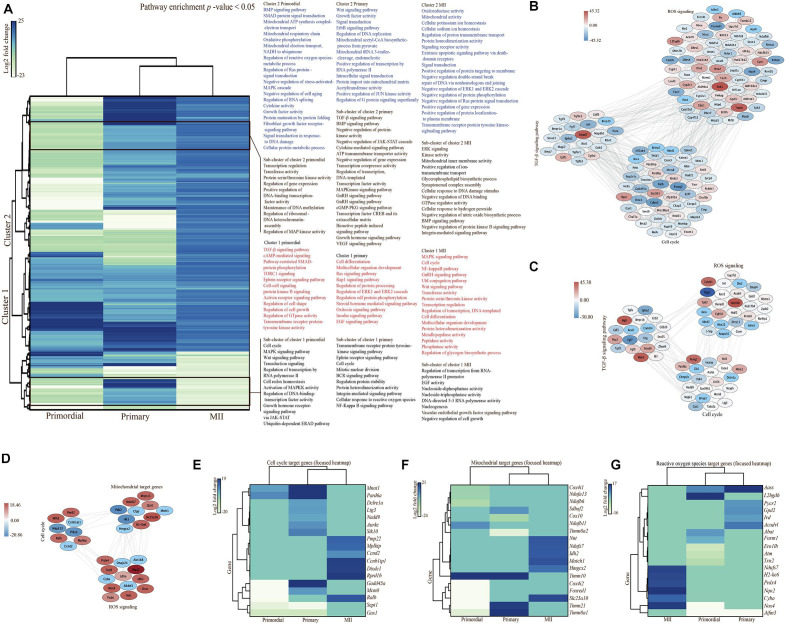
Pathway enrichment analysis in oocytes. **(A)** Hierarchically clustered heatmap of the 302 selected significantly differentially expressed genes (SDEGs) in oocytes from primordial and primary follicles and MII oocytes in the *in vitro* group compared with the *in vivo* group. Pathway enrichment analysis was performed in each cluster of genes. Each subcluster of clusters is outlined, and the list of significantly enriched pathways is shown for each cluster and subcluster. Columns show different developmental stages of oocytes at the bottom. The color code based on log2 fold changes is shown as a scale (ranging from –23 to 25). **(B–D)** Interaction networks are shown for differentially expressed genes associated with the TGF-β signaling pathway, cell cycle, and ROS in oocytes from primordial **(B)** and primary follicles **(C)** and cell cycle-, ROS-, and mitochondria-related genes in MII oocytes **(D)**. **(E–G)** Focused heatmaps of the cell cycle **(E)**, mitochondrial function **(F)**, and ROS **(G)** of oocytes from primordial and primary follicles and MII oocytes in the *in vitro* group compared with the *in vivo* group. All involved genes are shown on the right-hand side, according to their colors on the left-hand side in each row of each heatmap. The color codes based on log2 fold changes are shown as a scale for each heatmap.

A PPI network was built from all SDEGs in MII oocytes (Figure 6E). Different expression patterns were observed between *in vivo-* and *in vitro*-grown MII oocytes and oocytes from primordial and primary follicles. Interestingly, a hierarchically clustered heatmap showed a major difference between MII oocytes and oocytes from both primordial and primary follicles based on the involved pathways in a comparison of the *in vivo* and *in vitro* groups (Figure 7A). In MII oocytes from the *in vivo* and *in vitro* groups, the expression genes in cluster 1 and cluster 2, as well as a subcluster of genes, were highly upregulated. Consistent with stage-specific observations of SDEGs, cell cycle- and mitochondria-related genes were found to have different expression patterns between *in vivo-* and *in vitro*-matured MII oocytes and oocytes from primordial and primary follicles (Figures 7B–D). Moreover, pathway enrichment analysis of SDEGs showed that most of the involved pathways, including the cell cycle, MAPK signal transduction, Wnt signaling, and calcium signaling pathways, were highly enriched in the *in vitro-*matured oocytes compared with the *in vivo*-matured MII oocytes (Figure 7A). The enrichment pathway analysis further showed that different signaling pathways such as bone morphogenetic protein (BMP) signaling, fibroblast growth factor (FGF) signaling, target of rapamycin complex 1 (TORC1) signaling, TGF-β signaling pathway, mitochondrial function, and ROS signaling, in oocytes from primordial follicles, were changed during *in vitro* culture condition in oocytes from primordial follicles during *in vitro* culture. Specifically, in oocytes from primary follicles, several signaling pathways such as vascular endothelial growth factor (VEGF) signaling, epidermal growth factor (EGF) signaling, TGF-β signaling pathway, MAPK signaling transduction, cell cycle, mitochondrial function, and ROS signaling were altered during *in vitro* culture. In addition, extracellular signal-regulated kinase (ERK) signaling, gonadotropin releasing hormone (GnRH) signaling, ubiquitylation (UbI) conjugation pathway, nuclear factor kappa-light-chain enhancer of activated B cell (NF-KappaB) pathway, cell cycle, mitochondrial function, and ROS signaling in MII oocytes were changed during *in vitro* culture. In conclusion, the results showed that there was a large difference between early and late stages of follicular development and oocyte maturation, in relation to signaling pathways.

Curiously, expression of the majority of genes in subcluster 1 and cluster 2, as well as its subcluster of genes, was downregulated in oocytes from primordial follicles from the *in vitro* group compared with the *in vivo* group (Figure 7A). In contrast, expression of the majority of genes of both clusters 1 and 2, as well as their subclusters of genes, was upregulated in oocytes from primary follicles in the *in vitro* group compared with the *in vivo* group. Pathway enrichment analysis of gene sets from the clusters showed that genes involved in the cell cycle, TGF-β signaling pathway, MAPK signaling transduction, mitochondrial function, and ROS signaling were mostly enriched in oocytes from primary follicles. These results were consistent with our SDEG data showing a large fold change in volcano plots. To examine the TGF-β signaling pathway, cell cycle, ROS signaling, and mitochondria, we generated networks of SDEGs involved in these functions in oocytes from both primordial and primary follicles and MII oocytes (Figures 7B–D). Intriguingly, a different gene expression pattern in the TGF-β signaling pathway was noted between the *in vivo-* and *in vitro*-grown oocytes from primordial (Figure 7B) and primary follicles (Figure 7C), suggesting follicle stage-specific TGF-β-related functions. Notably, cell cycle- and ROS-related genes displayed different follicle-stage expression patterns in oocytes grown *in vivo* and *in vitro* (Figures 7B,C). For more details, focused heatmaps were generated for cell cycle (Figure 7E), mitochondria-related (Figure 7F), and ROS-related (Figure 7G) target genes, which revealed down- or upregulation of the expression of the genes in the *in vitro* oocytes compared with *in vivo* oocytes from primordial follicles and MII oocytes.

## Discussion

### A Resource for Elucidating the *in vitro* Activation and Maturation of Ovarian Follicles

In the present study, we provided detailed insights into stage-specific gene expression profiles and the corresponding signaling pathways of activated and mature mouse ovarian follicles grown either *in vivo* or *in vitro* by analyzing transcriptomes from oocytes from primordial and primary follicles and comparing them with those of MII oocytes. Determination of the detailed transcriptomes of follicle stage-specific oocytes *in vivo* and *in vitro* will contribute to *in vitro* growth and be a valuable source for future optimization of fertility treatments. Excitingly, we identified genes encoding signaling factors related to the cell cycle, mitochondria, and ROS production. We identified approximately 1,000–3,000 DEGs in oocytes from primordial and primary follicles in relation to their *in vivo* and *in vitro* conditions and approximately 300 DEGs in *in vivo-* and *in vitro*-matured MII oocytes. Despite the fact that oocytes grown *in vivo* and *in vitro* in different developmental follicle stages showed similar morphology and quality, our transcriptomic analysis identified different molecular characteristics due to the *in vitro* culture system.

### *In vivo* and *in vitro* Growth of Oocytes From Primordial and Primary Follicles

Surprisingly, we detected major changes in the mechanisms other than classic oocyte-activating PI3K–PTEN–AKT–FOXO3 signaling, which may elucidate the mechanisms underlying follicular development. Our functional analysis indicated that one of the dominant processes affected by *in vitro* conditions was cell cycle progression. Interestingly, most cell cycle-related genes, such as *Cdk10*, *Ogfr*, and *Ralb* and *Psmg2* and *Cyren*, were among the top 20 downregulated DEGs in oocytes from primordial follicles. *Cdk10* is a Cdc2-related kinase during the G2 or M phase and promotes cell division (Zehra et al., 2019). Moreover, *Cdk10* regulates transcriptional activity and development through the Raf–MEK–ERK1/2 pathway, showing its role in development as well as the cell cycle (Guen et al., 2017). Interestingly, several cell cycle activator (*Cdk10*, *Ralb*) and inhibitor (*Ogfr*, *Psmg2*, *Cyren*) genes were differentially expressed in *in vitro*-grown oocytes compared with *in vivo*-grown oocytes from primordial follicles.

Interestingly, downregulation of the expression of *Cdk10*, *Cyren*, and *Psmag2*, which are regulators, and *Ogfr*, a suppressor, and *Ralb*, as an activator of the cell cycle, suggests a possible fine-tuning balance between these activators and suppressors of cell cycle progression during *in vitro* culture.

Surprisingly, despite downregulation of the expression of most DEGs associated with the cell cycle in oocytes from primordial follicles, oocytes from primary follicles showed upregulation of the expression of most cell cycle-associated genes, such as *Cyren*, *Psmg2*, and *Mnat1*. Surprisingly, the expression of *Cyren* and *Psmg2*, which showed strong downregulation in oocytes from primordial follicles, was highly upregulated in oocytes from primary follicles in the present study. These results suggested that there may be a dynamic pattern in cell cycle function from primordial to primary follicle development, which was changed during *in vitro* culture, showing the key role of cell cycle-involved genes in the primordial to primary follicle transition. This finding suggested the important role of regulating the cell cycle in the activation of dormant follicles. Accordingly, differential expression of genes associated with the cell cycle and growth was detected in previous studies in mice (Dharma et al., 2009), rats (Kezele et al., 2005), humans (Markholt et al., 2012), and primates (Arraztoa et al., 2005), along with global analysis of murine follicles (Chang et al., 2016) and microarray analysis of human and bovine oocytes (Adona et al., 2016; Ye et al., 2020).

In recent studies, members of the TGF-β superfamily have been investigated due to their regulatory role in ovarian function and fertility (Galloway et al., 2000; McNatty et al., 2005; Li, 2015; Gao Y. et al., 2017). Notably, our pathway analysis suggests that the TGF-β signaling pathway, e.g., TGF-β-associated genes (such as *Pura*, *Snx25*, and *Smad7*), regulates ovarian *in vitro* culture. We found that the expression of several genes associated with the TGF-β signaling pathway (such as *Pura* and *Snx25*) was downregulated in oocytes from primordial follicles but enriched in oocytes from primary follicles, indicating that the TGF-β signaling pathway plays a role in primordial follicle activation during *in vitro* culture. Interestingly, *Pura* and *Snx25* expression was highly downregulated in oocytes from primordial follicles. *Snx25* is a regulator of the TGF-β signaling pathway that can increase the degradation of TGF-β receptors by the lysosomal pathway, and overexpression of *Snx25* suppressed TGF-β activity, while knockdown of endogenous SNX25 expression increased the level of TGF-β receptors and enhanced TGF-β signaling (Hao et al., 2011). Therefore, strong downregulation of *Snx25* expression in oocytes from primordial follicles showed that *in vitro* culture affects primordial follicle activation by suppressing the expression of this gene, leading to increased TGF-β signaling. Despite downregulation of the expression of *Snx25*, an inhibitor of TGF-β signaling, *Pura* expression was also highly downregulated in the *in vitro* oocytes compared with *in vivo* oocytes from primordial follicles and may activate TGF-β signaling. *Pura* encodes a multifunctional DNA- and RNA-binding protein. A previous study showed that PURA binding to TGF-β led to increasing TGF-β1 promoter activity in astrocytic cells (Thatikunta et al., 1997). However, the roles of SNX25 and PURA and their regulation of the TGF-β signaling pathway in oocyte development are unknown.

In addition, we showed that one of the top 20 significantly upregulated genes in *in vitro-* compared with *in vivo*-grown oocytes from primordial follicles was *Smad7*, which plays a regulatory role in a negative feedback loop for TGF-β signaling. SMAD proteins are downstream signaling transducers of the TG-Fβ superfamily (Gao et al., 2013). *Smad7* plays a regulatory role in a negative feedback loop for TGF-β signaling. SMAD7 competes with SMAD2/3 to bind to a type I TGF-β receptor and regulate TGF-β signaling (Quezada et al., 2012).

We also showed strong upregulation of the expression of several other TGF-β superfamily associated genes, such as *Ngfr*, *Smad6*, *Fgf9*, *Pitx2*, and *Snx25*, in oocytes from primary follicles. *Ngfr* encodes nerve growth factor receptor (NGFR), which is a multifunctional cell surface receptor and is expressed in several cell types (Bao et al., 2020). Although *Ngfr* is known for its function in nervous system development, a previous study described *Ngfr* expression and its critical role in regulating the growth, development, and function of reproductive organs (Bu et al., 2016). Moreover, a recent study identified *Ngfr* expression in follicles of different sizes in porcine ovaries during estrus, suggesting its essential role in the function of the ovary in sows (Jana et al., 2011). *Ngfr* expression was also detected in granulosa cells, thecal cells, stromal cells, and luteal cells of goat ovaries (Ren et al., 2005). Moreover, *Smad6*, one of the top 20 upregulated DEGs between *in vivo* and *in vitro* oocytes from primary follicles, regulates TGF-β signaling and can suppress BMP signaling. SMAD6, which protects oocytes, can govern TGF-β signaling and prevent overactivation, affecting reproductive function. Deletion of *Smad6* in the ovary affected cell cycle gene expression and resulted in abnormal granulosa cell proliferation, differentiation, and apoptosis through overactivation of TGF-β signaling (Li, 2015). Upregulation of the expression of *Smad7* in oocytes from primordial follicles and *Smad6*, as regulators of TGF-β signaling, in oocytes from primary follicles indicates their distinct regulatory roles in each developmental stage of follicles.

In addition, the expression of *Fgf9*, which showed upregulation between *in vivo-* and *in vitro-*grown oocytes from primary follicles, is a member of the FGF family and is involved in ovarian follicular development. The role of FGF9 in steroidogenesis in the ovary has been shown to be dependent on species (Schutz, 2016). Previous studies suggested a link between TGF-β and FGF signaling through regulation of FGF signaling by TGF-β signaling (Mukherjee et al., 2005; Strand et al., 2014; Bordignon et al., 2019). Moreover, previous studies reported that *Fgf9* can enhance the expression of *Pitx2* (Bellusci, 2008; Geske et al., 2008), which unexpectedly was one of the top upregulated genes in oocytes from primary follicles in the present study. Different roles of *Pitx2* in cell proliferation, differentiation, and organogenesis have been reported in several studies (Lin et al., 1999; Liu et al., 2001; Ai et al., 2006; Hayashi et al., 2008). *Pitx2* can interact with β-catenin to regulate cell proliferation by inducing the transcription of cyclin Ds and c-Myc (Yoshioka et al., 1998; Iwao et al., 2009). A recent study on neural crest cells showed that *Pitx2* acts downstream of TGF-β and FGF signaling and regulates cell proliferation through induction of cyclin D1 and D3 gene expression (Iwata et al., 2012). Therefore, taken together with the other studies, our data showed the strong upregulation of *Fgf9* and *Pitx2* expression along with upregulation of the expression of genes involved in the cell cycle in oocytes from primary follicles in a comparison between *in vivo* and *in vitro* conditions in this study, which may indicate the role of the TGFβ–*Fgf9*–*Pitx2* signaling cascade in ovarian follicular activation. Interestingly, our transcriptomic analysis showed overlapping significant differential expression of *Snx25* between oocytes from primordial and primary follicles with slight functional interconnections in oocytes from both primordial and primary follicles in constructed networks from SDEGs. Surprisingly, *Snx25* expression was highly upregulated in oocytes from primary follicles but highly downregulated in oocytes from primary follicles, suggesting a role in primordial follicle activation and development, which was changed through *in vitro* culture. These results showed a possible dynamic pattern in TGF-β signaling from primordial to primary follicle development, suggesting the importance of these genes in the TGF-β signaling pathway in the primordial to primary follicle transition.

The main source of ROS production is the mitochondria, and as expected, we noted major changes in mitochondria-related genes between the *in vivo* and *in vitro* groups. However, the expression of most ROS-related genes, such as *Dcxr*, *Foxred1*, *Nox4*, and *Tph2*, was downregulated in oocytes from primordial follicles. In contrast, the expression of ROS-related proteins, such as *Akr1b8*, *Cybrd1*, and *Tph2*, was upregulated in oocytes from primary follicles. Therefore, mitochondria-related genes may lead to an increase in mitochondrial function, resulting in the enrichment of genes involved in ROS signaling in oocytes from primary follicles. Interestingly, *Nox4*, as a member of the NOX family in NADPH oxidases, is widely expressed in different types of cells and is the main source of ROS. A recent study reported that NOX4-derived ROS production increased granulosa cell proliferation and promoted follicle growth (Buck et al., 2019). *Nox4* was also shown to induce the expression of MAPK family members and affect the signal transduction system (Buck, 2018). In addition, previous studies indicated that *Nox4* is related to cell death and increases apoptosis (Seo et al., 2017; Yang et al., 2018). Therefore, our results showed that *in vitro* culture may decrease intracellular ROS production through downregulation of *Nox4* expression in oocytes from primordial follicles. In contrast, the expression of *Dcxr*, which has a protective role against oxidative stress, was most significantly downregulated in oocytes from primordial follicles in the present study, suggesting a possible balance between the expression of ROS generators and detoxifying genes in oocytes from primordial follicles during *in vitro* culture. *Dcxr* is critical for carbonyl detoxification and carbohydrate metabolism (Yang et al., 2017). However, the function of *Dcxr* is controversial. Despite the protective role of *Dcxr* in decreasing oxidative stress through detoxification of reactive carbonyls, its ability to generate ROS by the redox cycling of 9,10-phenanthrenequinone was reported in previous studies (Ebert et al., 2015).

Overall, we found several genes related to mitochondrial function and ROS production in oocytes from both primordial and primary follicles, suggesting the effect of *in vitro* culture on mitochondrial function and ROS levels. However, downregulation of the expression of mitochondrial and ROS-related genes in oocytes from primordial follicles and enhancement of these functions in oocytes from primary follicles showed that oocytes require more energy, which granulosa cells cannot supply during primordial follicle activation in culture, leading to the induction of mitochondrial function and subsequent ROS production.

### *In vivo* and *in vitro* Maturation of MII Oocytes

We used a 3D culture system to determine whether different molecular characteristics affect oocyte quality and maturation to the MII stage. We observed that oocytes from two-step-cultured ovaries successfully matured to the MII stage, and their morphology, as well as their mitochondrial function and ROS levels, was similar to that of *in vivo*-matured oocytes, while their molecular characteristics were significantly different. Interestingly, our comparative transcriptomic analysis showed that the levels of most DEGs in the *in vivo-* and *in vitro*-matured MII oocytes were highly upregulated, which was different from oocytes from primordial follicles, which showed mostly downregulation of DEG expression. Accordingly, previous studies reported that the levels of most DEGs were upregulated in mouse and human oocytes after *in vitro* maturation, from the germinal vesicle (GV) to MII stage, compared with *in vivo*-matured oocytes (Jones et al., 2008; Gao L. et al., 2017; Liu et al., 2017). Interestingly, we found a major upregulation of the expression of most genes associated with the cell cycle, such as *Ccnb1ip1*, *Mlh3*, *Ralb*, *Piwil2*, and *Cyren*, between the *in vivo-* and *in vitro*-matured MII oocytes. Accordingly, previous studies reported that cell cycle-related genes were highly expressed in *in vitro*-matured oocytes compared with *in vivo*-matured oocytes, suggesting abnormalities in the cell cycle process in *in vitro*-matured oocytes (Wells and Patrizio, 2008; Ye et al., 2020). Surprisingly, in our transcriptomic analysis, *Ccnb1ip1* was the top upregulated DEG in MII oocytes. In mice, *Ccnb1ip1* encodes cyclin B1 interacting protein 1, a putative ubiquitin E3 ligase. Defects in *Ccnb1ip1* expression led to meiotic impairments in mice (Yoshida et al., 2015). In humans, the CCNB1IP1 protein is a RING finger family ubiquitin ligase that governs the G2/M phase in the cell cycle through degradation of cyclin B (Toby et al., 2003). Strong upregulation of *Ccnb1ip1* expression in MII oocytes cultured *in vitro* compared with *in vivo* suggested that *in vitro* culture affects resumption of meiosis in MII oocytes, which is critical for nuclear maturation.

Mitochondrial function has long been investigated and identified as a marker for oocyte quality due to its role in oocyte maturation (Gao L. et al., 2017; Ye et al., 2020). Interestingly, the levels of several mitochondria-related genes, such as *Mtch1*, *Ndusf7*, *Mrps11*, and *Slc25a10*, were highly upregulated in the *in vitro*-matured MII oocytes compared with the *in vivo*-matured MII oocytes. Thus, *in vitro* culture may affect oocyte quality through enrichment of mitochondrial genes. Combined with other studies, these results showed that *in vitro* culture may change mitochondrial function and increase ATP production, which is needed under culture conditions. However, these studies are controversial. It was reported that the expression of genes involved in the mitochondrial respiratory chain in bovine *in vitro*-matured oocytes was downregulated compared with that in *in vivo*-matured oocytes, suggesting negative effects of *in vitro* culture on mitochondrial function and oocyte maturation (Katz-Jaffe et al., 2009). These discrepancies may be due to differences in culture methods and species. Therefore, upregulation of the expression of mitochondria-related genes in MII oocytes in the present study suggested a higher demand for energy in mature oocytes, which is required to prepare oocytes for fertilization and subsequent embryonic development during *in vitro* culture.

Reactive oxygen species, which are produced during *in vitro* maturation of oocytes, are known to decrease oocyte quality (Soto-Heras and Paramio, 2020). Therefore, we compared the ROS levels in the *in vitro*-matured MII oocytes with those from the oocytes matured *in vivo* and found no difference between the groups. Thus, the *in vitro* culture appears not to affect the ROS levels of MII oocytes. However, it is important to note that the ROS measurements using DCFH-DA are associated with restrictions regarding evaluation of cellular oxidative stress. This is due to its hydrophilic nature, preventing all DCFH-DA to cross the membrane, and its dependence on Fenton-type reactions and/or on unspecific enzymatic oxidation by cytochrome *c* (Karlsson et al., 2010). A study showed that after 12 h of incubation for *in vitro* maturation of bovine oocytes, ROS levels were enhanced (Morado et al., 2009). Moreover, several studies in mice, pigs, and cows demonstrated that low oxygen tension with low levels of ROS production led to successful blastocyst formation (Hashimoto et al., 2000; Iwamoto et al., 2005). In contrast, high oxygen tension improved *in vitro* maturation of porcine oocytes and enhanced the blastocyst rate (Park et al., 2005). Furthermore, oxygen tension, which can increase ROS production, did not change the nuclear and cytoplasmic maturation of bovine oocytes during *in vitro* maturation (Giotto et al., 2015). Examination of different oxygen concentrations during mouse follicle *in vitro* maturation showed that increasing oxygen concentrations led to high levels of ROS production, which did not affect maturation, fertilization, and early developmental rates; however, the numbers of blastocysts were significantly reduced (Banwell et al., 2007). Therefore, further in-depth research is needed to determine the effect of *in vitro* conditions and ROS production on oocyte quality. Surprisingly, despite similar ROS levels in the *in vivo-* and *in vitro*-matured MII oocytes, our functional pathway analysis showed that genes involved in ROS signaling, such as *Nqo2*, *Prdx4*, *Dnajc24*, *Nox4*, *Dcxr*, and *Akr1b8*, were enriched in MII oocytes. These results suggested that *in vitro* culture may lead to major changes in ROS production-related genes in MII oocytes from *in vitro* maturation compared with *in vivo* maturation, while the ROS level of the *in vitro*-matured MII oocytes was not significantly different from that of the *in vivo*-matured oocytes. However, as the main source of intracellular ROS is the mitochondria and mitochondrial dysfunction can lead to an increase in ROS levels, enrichment of mitochondria-related genes in MII oocytes may cause changes in ROS-related genes between the *in vivo-* and *in vitro*-matured MII oocytes.

These results indicated that significant differences in the expression of mitochondria-related genes can result in alteration of mitochondrial function, leading to major changes in ROS signaling in MII oocytes, as well as oocytes from primordial and primary follicles. However, although there were major changes in ROS signaling in oocytes from different developmental stages in the present study, our results suggest a transcriptional regulation of genes involved in ROS generation and related to antioxidant function in MII oocytes as well as oocytes from primordial and primary follicles.

## Conclusion

In summary, we showed that *in vitro* culture conditions changed cellular functions that were not related to the classic follicle-regulating AKT/PI3K/PTEN pathway in oocytes at different developmental stages. Alterations in these functions during the culture period suggested their important role in follicular development and oocyte maturation. We found various candidate genes in these signaling pathways, which have not been well studied in oocytes, and their role in oocyte development is unknown. Our comparative transcriptomic analysis showed a fluctuation in the expression of genes between oocytes from different developmental stages, suggesting a possible dynamic pattern of different functions in oocytes from early to late developmental stages. Therefore, we concluded that the important transcriptomic drivers that activate *in vivo* primordial oocytes are involved in the cell cycle, TGF-β signaling, MAPK signal transduction, Wnt signaling, and mitochondrial function. Although we identified overlapping differentially expressed genes in oocytes from primordial and primary follicles, the transcriptomes of MII oocytes showed significant divergence. These data will increase our understanding of ovarian follicle development and activation to help restore and preserve female fertility (Niu and Spradling, 2020; Hamazaki et al., 2021).

## Materials and Methods

### Animals

Female C57BL/6JRj mice and male CBA/JRj mice were housed under a 12-h light/dark cycle and bred to produce C57BL × CBA F1 mice in the biomedical animal facilities at Aarhus University. All procedures were approved by the Ethics Committee for the Use of Laboratory Animals at Aarhus University (2015-15-0201-00800 to KL-H).

### Experimental Design

The ovaries from 7- to 14-day-old F1 (C57Bl/6j × CBA/J) female mice were removed after cervical dislocation. Then, ovaries were dissected from their surrounding tissues and washed with alpha-minimal essential medium (α-MEM, Gibco, Scotland, United Kingdom) supplemented with 10% fetal bovine serum (FBS, Gibco). The ovaries from 7-day-old female mice were cultured for 7 days. Then, some of the cultured ovaries (*in vitro* group) and non-cultured ovaries from 14-day-old mice (*in vivo* group) were fixed, dehydrated, embedded, and stored at –80°C for morphological assessment and RNA sequencing. Another group of cultured ovaries was used for isolation and culture of preantral follicles for an additional 12 days. Then, the collected MII oocytes were used for ROS assays, mitochondrial membrane potential assays, and RNA sequencing and compared with derived MII oocytes (Figure 1B).

### Encapsulation and 3D Culture of Isolated Preantral Follicles

The secondary follicles (*n* > 150) from cultured ovaries were isolated and encapsulated using sodium alginate as previously defined. Briefly, sodium alginate was dissolved in deionized water at a concentration of 1% (w/v) and filtered. After each follicle was transferred in a droplet of sodium alginate (7 μl), the droplets were placed in a cross-linking solution (50 mM CaCl_2_ and 140 mM NaCl). Next, the alginate beads were transferred to α-MEM medium for rinsing. Encapsulated follicles were cultured in α-MEM supplemented with 5% FBS; 100 mIU/ml recombinant follicle stimulating hormone (rFSH) or GONAL-f (Serono, Geneva, Switzerland); 1% insulin, transferrin, and selenium (ITS; Gibco); 100 mg/ml penicillin; and 50 ng/ml streptomycin under mineral oil at 37°C with 5% CO_2_ for 12 days. During the culture period, half of the culture media was replaced with fresh media every other day.

### Diameter of Follicles

The mean diameters of follicles and oocytes were measured for each follicle in both groups to determine follicle growth dynamics. Images of follicles from the *in vivo* and *in vitro* groups at the primordial and primary stages (*n* = 50 per ovary in each group at each stage) were taken using an inverted microscope with an attached digital camera (Leica, BMI4000B, Wetzlar, Germany). ImageJ software (National Institutes of Health, Bethesda, MD, United States) was used to calculate the diameter of follicles and oocytes in micrometers. Two perpendicular diameters from the basement membrane to the basement membrane in follicles and from the plasma membrane to the plasma membrane in oocytes were used to calculate the mean diameters.

### Immunofluorescence Analysis

The embedded ovaries in each group were sectioned (5 μm thickness) and rehydrated. After antigen retrieval at 95°C for 15 min, permeability was enhanced in 0.5% Triton X-100 in phosphate-buffered saline (PBS) for 10 min. Then, the slides were blocked using 10% normal donkey serum (Chemicon^®^, S30, Temecula, CA, United States) in PBS for 30 min. Next, the slides were incubated overnight at 4°C with FOXO1, FOXO3a, PTEN, and mTOR antibodies at 1:100, 1:200, 1:50, and 1:100 dilutions in PBS containing 10% donkey serum. The slides were incubated in a 1:300 dilution of secondary antibody (donkey-anti-rabbit) conjugated with Alexa Fluor 488 dye (Thermo Fisher Scientific, Waltham, MA, United States), followed by incubation in 1/1,000 DAPI (Sigma, Saint Louis, MO, United States). The slides were then mounted using Dako Fluorescent Mounting Medium (Agilent Technologies, Santa Clara, CA, United States) and assessed using an LSM510 laser-scanning confocal microscope using a × 63 C-Apochromat water immersion objective NA 1.2 (Carl Zeiss, Göttingen, Germany). The images were captured and analyzed using Zen 2011 software (Carl Zeiss).

### ROS Assay

The collected MII oocytes from the *in vivo* and *in vitro* groups (*n* = 60 for each group in three repeats) were pooled and transferred to PBS for washing. Next, they were incubated in 40 mmol/L Tris–HCl buffer (pH = 7.0) containing 5 mmol/L DCFH-DA (Sigma) at 37°C for 30 min. After the samples were washed in PBS, they were sonicated at 50 W for 2 min. They were then centrifuged at 40°C and 10,000 × *g* for 20 min and analyzed using a spectrofluorometer at 488 nm excitation and at 525 nm emissions.

### Mitochondrial Membrane Potential Assay

The integrity of the inner mitochondrial membrane was assayed by measuring the uptake of the cationic carbocyanine dye JC-1 (5,5′,6,6′-tetrachloro-1,1′,3,3′-tetraethylbenz-imidazolcarbocyanine iodide) into the matrix with the formation of J-aggregates according to the protocol described in the MITO-ISO1 kit (Sigma-Aldrich). Sample fluorescence was monitored in a spectrofluorometer at 590 nm emission and 490 nm excitation. The fluorescence produced was calculated per mg mitochondrial protein (FLU/mgP).

### Culture of Ovaries

The dissected ovaries from 7-day-old female mice were cultured on inserts (pore size of 0.4 μm, 6.5 mm diameter, Corning, MA, United States) in 24-well plates at 37°C and in a humidified atmosphere of 5% CO_2_–95% air. The culture medium was α-MEM supplemented with 10% FBS; 5 μg/ml insulin, 5 μg/ml transferrin, and 5 ng/ml sodium selenite (1 × ITS; Gibco Cat. No. 41400045); 1 × penicillin–streptomycin solution (Thermo Fisher Cat. No. 15140122); and 100 mIU/ml rFSH or GONAL-f (Serono).

### Histological Evaluation

The ovaries in each group (*n* = 5/group) were fixed in 4% paraformaldehyde, dehydrated, and embedded in paraffin. Then, they were serially sectioned into 5-μm-thick slices and stained using hematoxylin and eosin (H&E). The follicles were counted in every fifth section of each ovary, and the follicles with nuclei were used for counting to avoid duplicate counting in each ovary. The classification of follicles was defined previously. Briefly, follicles with flattened pregranulosa cells surrounding the oocyte were defined as primordial follicles. Moreover, follicles with a single layer of cuboidal granulosa cells were considered primary follicles, and follicles with two or more granulosa cell layers were classified as secondary follicles. Normal follicles were defined as follicles with an intact oocyte and organized granulosa cells. In contrast, degenerated follicles were defined as follicles with shrunken ooplasm, pyknotic oocyte nuclei, and disorganized granulosa cells. The percentage of follicles in each developmental stage was calculated based on the number of normal follicles from the *in vivo* (*n* = 5 ovaries, *n* = 1,314 primordial, *n* = 170 primary, and *n* = 440 secondary follicles) and *in vitro* (*n* = 5 ovaries, *n* = 1,152 primordial, *n* = 134 primary, and *n* = 438 secondary follicles) groups.

### *In vitro* and *in vivo* Ovulation Induction

Ovulation was induced by adding 1.5 IU/ml hCG (Serono) to the culture media after 12 days of follicular culture. The released MII oocytes were then collected as an *in vitro* group. Adult female mice (*n* = 9) were induced to superovulate by intraperitoneal (IP) injection of 10 IU human menopausal gonadotrophin (HMG, Folligon; Intervet, Noord-Brabant, Netherlands) followed by injection of 10 IU hCG 48 h later. Fresh MII oocytes, as an *in vivo* group, were then collected.

### Laser-Capture Microdissection

Non-cultured and cultured ovaries were fixed using 4% paraformaldehyde at 4°C followed by dehydration, embedding in paraffin, and sectioning. Then, the sections were placed on membrane glass slides (Arcturus^®^ PEN Membrane Glass Slides, Applied Biosystems, Life Technologies, Foster City, CA, United States) and stained using H&E on the same day. Oocytes of primordial and primary follicles in both groups were isolated by LCM. Isolation of pure oocytes by LCM was based on morphological appearance. Oocytes surrounded by flattened pregranulosa cells were defined as oocytes from primordial follicles, and oocytes surrounded by one layer of cuboidal granulosa cells were defined as oocytes from primary follicles. An ultraviolet laser was used for cutting after marking a line around oocytes. Then, the samples were placed onto a sterile cap (Arcturus^®^ CapSure^®^ HS LCM Caps, Applied Biosystems, Life Technologies) using infrared pulses. The isolated cells were carefully monitored on the cap to avoid any contamination from surrounding granulosa cells.

### RNA Extraction

Arcturus^®^ Paradise^®^ Plus RNA Extraction and Isolation Kit (#KIT0312I Arcturus Bioscience Inc., Mountain View, CA, United States) was used for isolation of total cellular RNA from the microdissected cells on the LCM caps, according to the manufacturer’s protocol. RNA from each cap was pooled for the same oocyte stage during extraction. Total cellular RNA from oocytes collected *in vivo* and *in vitro* was isolated using Picopure (#KIT0204 Arcturus Bioscience, Inc.).

### Library Preparation and Sequencing

The Ovation^®^ PICO SL WTA System V2 RNA amplification system (#3312 NuGen, Inc., San Carlos, CA, United States) was used for conversion of isolated RNA from the LCM-derived samples to cDNA and linear amplification. Library preparation for microdissected oocytes was performed using an Illumina TruSeq RNA Access kit at BGI, according to the manufacturer’s protocol. RNA sequencing was performed on an Illumina HiSeq platform with two lanes (5 Gb per sample). Library preparation for collected oocytes in both groups was performed using the Smart-seq II method, and RNA sequencing was performed using the Illumina HiSeq platform with one lane (5 Gb per sample).

### Transcriptomic Analysis

The raw data were quality filtered and trimmed, and adaptor sequences were removed using trim_galore (v0.4.1). Quality control was performed using FastQC. Differential expression analysis was performed by mapping the filtered reads to the mouse genome (mm10) using TopHat2. Then, featureCounts software was used to quantify the number of reads mapped to each gene using gene annotation from Ensembl release 19. Differential expression analysis was performed using DESeq2 in R. Normalization was performed by DESeq2 to allow direct comparison between the samples. Then, for quick visualization and to reduce high-dimensional data to their most salient features, PCA was performed based on DEGs using R software (Figures 4B,E). Moreover, a quick visualization of adjusted *p*-values < 0.05 versus absolute fold change of 2 was performed using volcano plots, which were generated in R software (Figures 4C–F). For visualizing the SDEGs that overlapped between different developmental stages of oocytes, a Venn diagram was generated using BioVinci version 3.0.9 (Figure 5A). For visualizing overlapping expressed genes and their different linkages, the STRING database was used to create PPI networks in different developmental stages of oocytes, and the overlap between these oocytes was manually distinguished and marked in the networks (Figures 5B–D). For further and deep visualization, first, all SDEGs were shown, according to thresholds of an adjusted *p*-value of 0.05 and an absolute fold change of 2 (Figure 6A), followed by visualization of the full distribution in all SDEGs using a violin plot by BioVinci (Figure 6B). Second, 302 SDEGs were selected in all developmental stages of oocytes to focus on genes related to the hypothesis in the present study and unify in all stages. For further visualization of these selected genes, first, PPI networks were generated using the STRING database. Then, edges and nodes extracted from STRING were imported to Cytoscape version 3.8.2 (Figures 6C–E). For further visualization, a heatmap was generated by clustering the expression values with BioVinci. Then, pathway enrichment analysis for gene sets from each of the framed clusters in Figure 7A was performed using David Bioinformatic Resources version 6.8. Next, the focused networks were created using the STRING database and Cytoscape from manually annotated gene lists to visualize the TGF-β signaling pathway, cell cycle, and ROS signaling in oocytes from both primordial and primary follicles and cell cycle-, ROS-, and mitochondria-related genes in MII oocytes, which were visualized in a heatmap (Figures 7B–D). Finally, three focused heatmaps were extracted from the original heatmap to visualize specific genes related to the cell cycle, mitochondrial function, and ROS production (Figures 7E–G).

### Statistical Analysis

The follicular percentages, follicle diameters, ROS levels, and mitochondrial membrane potentials were analyzed by *t*-tests in SPSS version 27 software. Values are given as the mean ± SE. A *p*-value less than 0.05 was considered significant.

## Data Availability Statement

The data presented in the study are deposited in the Gene Expression Omnibus (GEO) repository under the accession code GSE179538 with the link: https://www.ncbi.nlm.nih.gov/geo/query/acc.cgi?acc=GSE179538.

## Ethics Statement

The animal study was reviewed and approved by all procedures were approved by the Ethics Committee for the use of laboratory animals at Aarhus University (2015-15-0201-00800 to KL-H).

## Author Contributions

Both authors contributed to this manuscript. MA and KL-H designed the study, interpreted and analyzed the data, and drafted the manuscript. MA performed the experiments.

## Conflict of Interest

The authors declare that the research was conducted in the absence of any commercial or financial relationships that could be construed as a potential conflict of interest.

## Publisher’s Note

All claims expressed in this article are solely those of the authors and do not necessarily represent those of their affiliated organizations, or those of the publisher, the editors and the reviewers. Any product that may be evaluated in this article, or claim that may be made by its manufacturer, is not guaranteed or endorsed by the publisher.

## Supplementary Material

Supplementary Figure 1 The fluorescence intensity of FOXO1, FOXO3a, PTEN, and mTOR in mouse ovaries from the *in vivo* and *in vitro* groups. There was no significant difference between the *in vivo* and *in vitro* groups. Values are given as the mean ± SE.

Supplementary Figure 2 Constructed protein–protein interaction network (PPI) from significantly differentially expressed genes (SDEGs) of oocytes from primordial follicles in the *in vitro* and *in vivo* groups. Red nodes represent the encoded proteins from overlapping SDEGs between oocytes from primordial and primary follicles in the *in vitro* and *in vivo* groups. Yellow nodes show encoded proteins from overlapping SDEGs between oocytes from primordial follicles and MII oocytes. Green nodes refer to encoded proteins from overlapping SDEGs between oocytes from all developmental stages.

Supplementary Figure 3 Constructed protein–protein interaction network (PPI) from significantly differentially expressed genes (SDEGs) of oocytes from primary follicles in the *in vitro* and *in vivo* groups. Red nodes represent the encoded proteins from overlapping SDEGs between oocytes from primordial and primary follicles in the *in vitro* and *in vivo* groups. Yellow nodes show encoded proteins from overlapping SDEGs between oocytes from primary follicles and MII oocytes. Green nodes refer to encoded proteins from overlapping SDEGs between oocytes from all developmental stages.

Supplementary Figure 4 Constructed protein–protein interaction network (PPI) from significantly differentially expressed genes (SDEGs) of MII oocytes in the *in vitro* compared to *in vivo* groups. Red nodes represent the encoded proteins from overlapping SDEGs between MII oocytes and oocytes from primordial follicles in the *in vitro* and *in vivo* groups. Yellow nodes show encoded proteins from overlapping SDEGs between MII oocytes and oocytes from primary follicles in the *in vitro* and *in vivo* groups. Green nodes refer to encoded proteins from overlapping SDEGs between oocytes from all developmental stages.
