# Supplementary figures and images for: Distinct Signaling Pathways Distinguish in vivo From in vitro Growth in Murine Ovarian Follicle Activation and Maturation
Source: Front Cell Dev Biol. 2021 Jul 23;9:708076. doi: 10.3389/fcell.2021.708076 (PMC8346253; doi:10.3389/fcell.2021.708076)

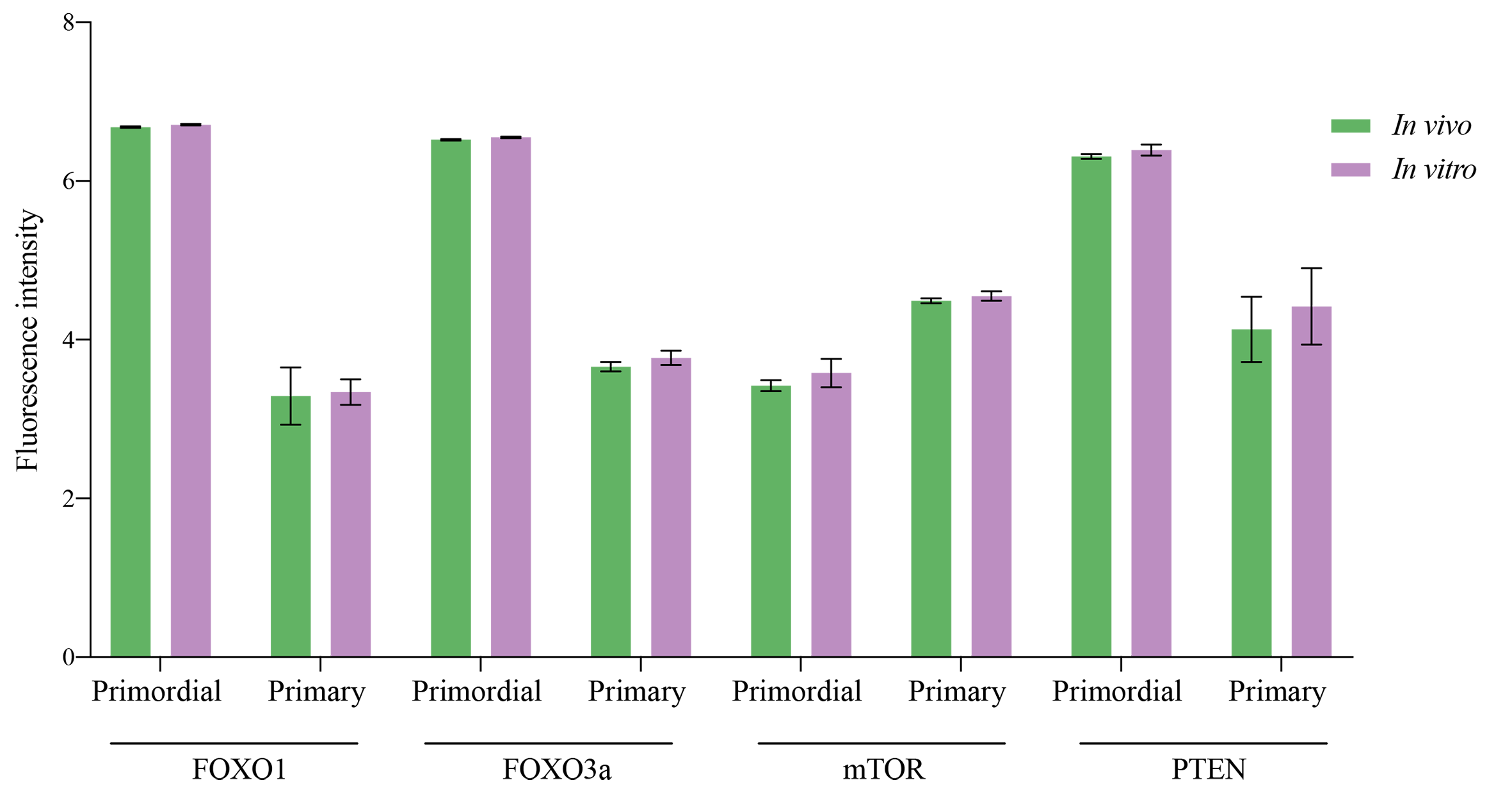

Supplement: Supplementary Figure 1 — The fluorescence intensity of FOXO1, FOXO3a, PTEN, and mTOR in mouse ovaries from the in vivo and in vitro groups. There was no significant difference between the in vivo and in vitro groups. Values are given as the mean ± SE. [file Image_1.tif]

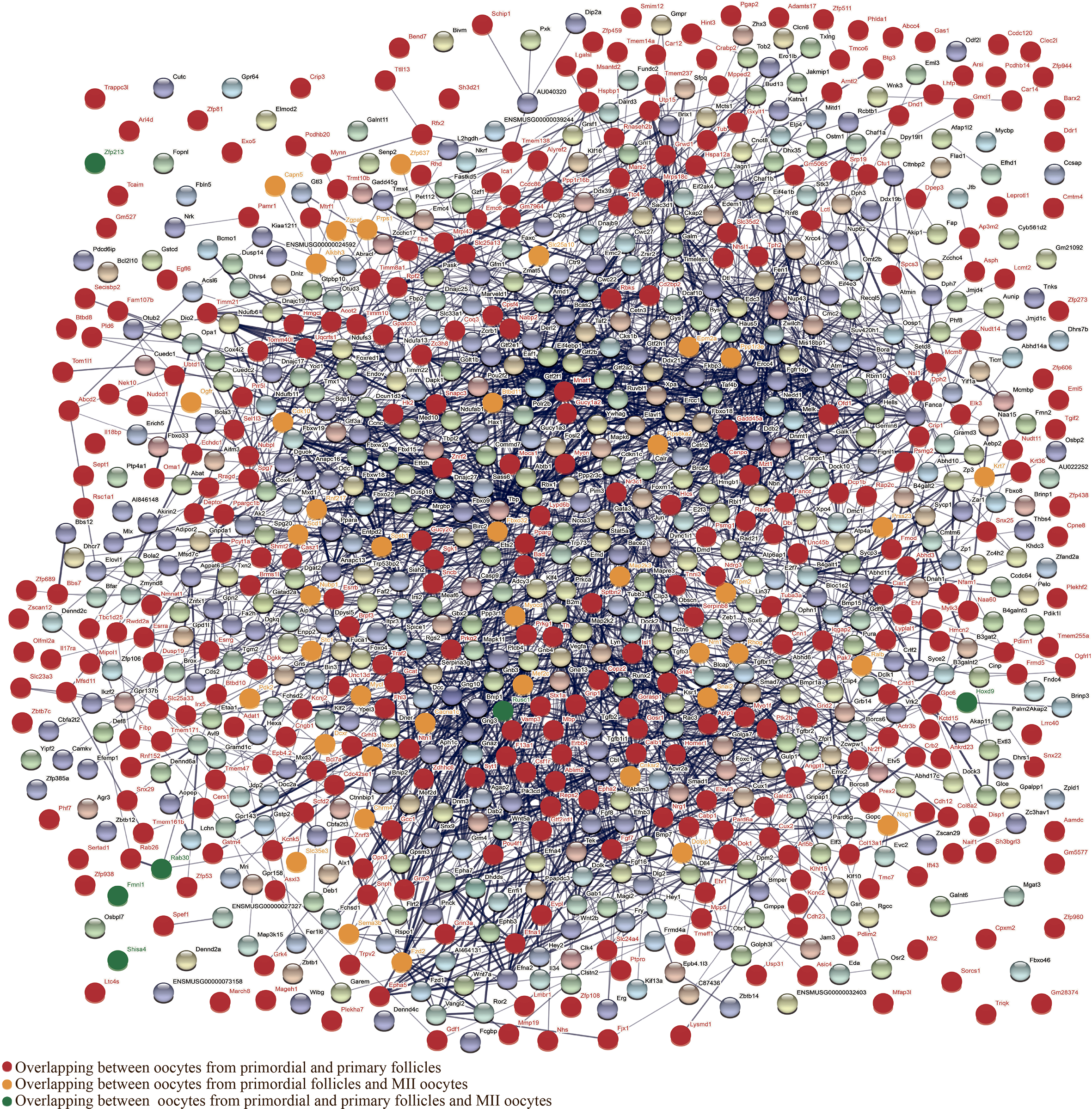

Supplement: Supplementary Figure 2 — Constructed protein–protein interaction network (PPI) from significantly differentially expressed genes (SDEGs) of oocytes from primordial follicles in the in vitro and in vivo groups. Red nodes represent the encoded proteins from overlapping SDEGs between oocytes from primordial and primary follicles in the in vitro and in vivo groups. Yellow nodes show encoded proteins from overlapping SDEGs between oocytes from primordial follicles and MII oocytes. Green nodes refer to encoded proteins from overlapping SDEGs between oocytes from all developmental stages. [file Image_2.tif]

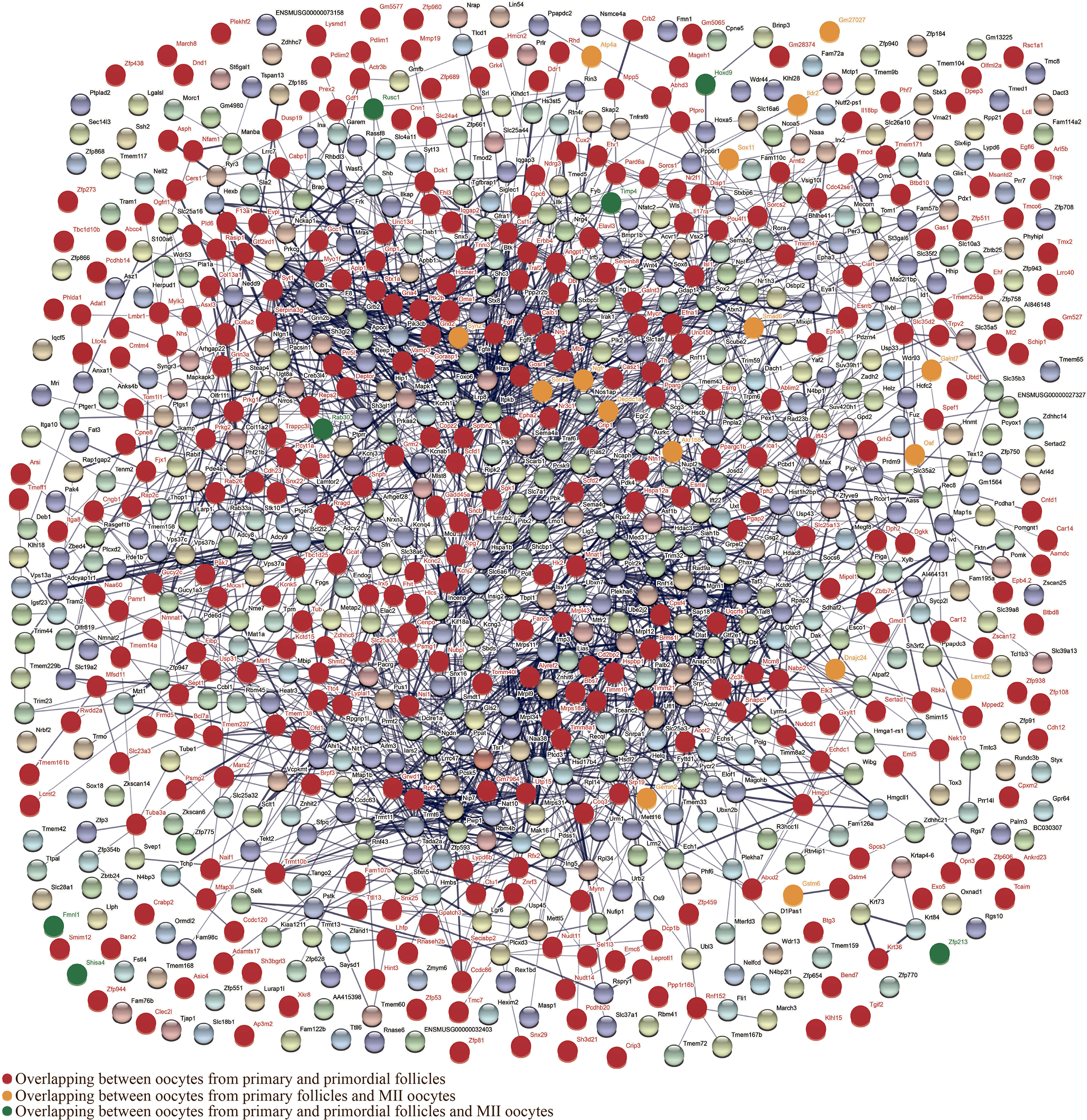

Supplement: Supplementary Figure 3 — Constructed protein–protein interaction network (PPI) from significantly differentially expressed genes (SDEGs) of oocytes from primary follicles in the in vitro and in vivo groups. Red nodes represent the encoded proteins from overlapping SDEGs between oocytes from primordial and primary follicles in the in vitro and in vivo groups. Yellow nodes show encoded proteins from overlapping SDEGs between oocytes from primary follicles and MII oocytes. Green nodes refer to encoded proteins from overlapping SDEGs between oocytes from all developmental stages. [file Image_3.tif]

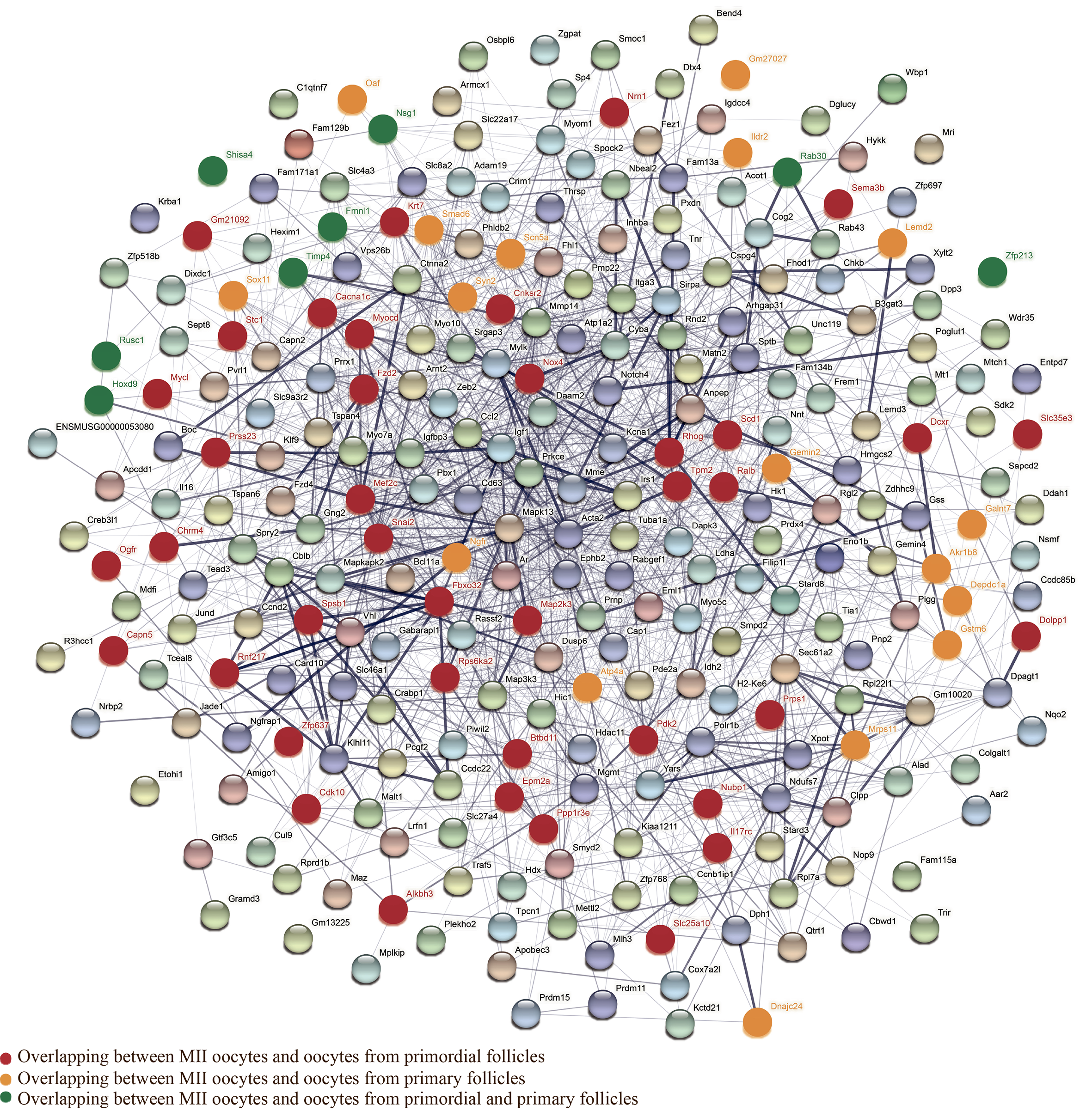

Supplement: Supplementary Figure 4 — Constructed protein–protein interaction network (PPI) from significantly differentially expressed genes (SDEGs) of MII oocytes in the in vitro compared to in vivo groups. Red nodes represent the encoded proteins from overlapping SDEGs between MII oocytes and oocytes from primordial follicles in the in vitro and in vivo groups. Yellow nodes show encoded proteins from overlapping SDEGs between MII oocytes and oocytes from primary follicles in the in vitro and in vivo groups. Green nodes refer to encoded proteins from overlapping SDEGs between oocytes from all developmental stages. [file Image_4.tif]
